# Redox therapy for neuropsychiatric disorders: Molecular mechanisms and biomarker development

**DOI:** 10.1126/sciadv.aea9014

**Published:** 2026-02-18

**Authors:** Kyle W. Cuklanz, Abigail Stein, Virginie-Anne Chouinard, Dost Ongur, Fei Du

**Affiliations:** ^1^ Psychotic Disorders Division, McLean Hospital, Belmont, MA 02478, USA.; ^2^ McLean Imaging Center, McLean Hospital, Belmont, MA 02478, USA.; ^3^ Harvard Medical School, Boston, MA, 02115, USA.; ^4^ Athinoula A. Martinos Center for Biomedical Imaging, Massachusetts General Hospital, Charlestown, MA 02129, USA.

## Abstract

Redox dysregulation, characterized by an imbalance in the NAD^+^ [nicotinamide adenine dinucleotide (oxidized form)]/NADH (reduced form of NAD^+^) ratio, is implicated in neurodegenerative and psychiatric disorders such as Alzheimer’s disease and schizophrenia. This imbalance contributes to mitochondrial dysregulation, oxidative stress, and inflammation. Despite promising preclinical studies supporting therapeutic strategies aimed at restoring redox balance and thereby rescuing brain bioenergetic deficits, clinical outcomes and efficacy remain limited. Progress has been hindered by the incomplete understanding of NAD^+^ subcellular cycling, as well as a lack of in vivo biomarkers measuring target engagement of redox status and mitochondrial function. Thus, this review examines molecular mechanisms of NAD (nicotinamide adenine dinucleotide)–related bioenergetic deficits, current and emerging NAD-targeted therapies, and recent advances in the development of neuroimaging biomarkers, emphasizing personalized and mechanism-driven approaches.

## Teaser

Redox therapy may restore brain energy deficits, underscoring the need for in vivo biomarkers to guide personalized treatments.

## INTRODUCTION

Alzheimer’s disease (AD) is a progressive neurodegenerative disorder that currently affects an estimated 6.7 million Americans (*1*). Despite substantial investment in AD research worldwide, the therapeutic efficacy of paradigms targeting the accumulation of misfolded amyloid and Tau remains limited. There is an urgent need to develop more effective methods of diagnosis and treatment (*2*). Treatments focusing on disrupted brain bioenergetics (i.e., energy metabolism) are especially promising, as mitochondrial dysregulation and cellular energy deficits are increasingly thought to play a critical role in aging and AD pathophysiology (*3*, *4*). In addition to AD, amyotrophic lateral sclerosis, Parkinson’s disease (PD), and cognitive aging (aging) are known to be characterized by metabolic deficits with mitochondrial dysregulation, pointing to a potential shared mechanism underlying neurodegenerative conditions (*5*).

Similarly, psychotic disorders, including schizophrenia (SZ) and bipolar disorder (BD), are common and severe, yet their pathophysiology remains poorly understood. Current treatments for psychotic disorders primarily target positive symptoms such as hallucinations and delusions (*6*), although negative and cognitive symptoms are increasingly considered to be key detriments to health and functioning (*7*). Consequently, many patients are partially or entirely unresponsive to available treatments and may suffer substantial side effects (*6*). Similar to AD, PD, and aging, emerging evidence suggests that psychotic disorders are associated with a host of bioenergetic abnormalities in the brain. Furthermore, cellular energy deficits may be a key contributor to negative and cognitive symptoms (*8*, *9*). A recent gene expression study suggested that AD and SZ may have some shared pathophysiological basis for cognitive impairment (*10*). The energy deficits observed in various neuropsychiatric disorders are often associated with redox dysregulation, specifically an imbalance between the oxidizing and reducing forms of nicotinamide adenine dinucleotide (NAD) [nicotinamide adenine dinucleotide (oxidized form) (NAD^+^) and reduced form of NAD^+^ (NADH)] (*5*, *11*–*13*).

Accumulating evidence suggests that therapeutic interventions targeting brain bioenergetics may ameliorate some of the clinical and neurological effects of neuropsychiatric disorders. NAD^+^ supplements have been shown to elevate blood NAD^+^ levels robustly (tables S10 to S13), and rodent studies repeatedly indicate that supplements can restore cellular redox balance, reduce oxidative stress, and enhance the brain’s mitochondrial function (*5*). In contrast, clinical studies have produced mixed results (tables S1 to S6), and some studies have been criticized for overstating their significance (*14*). However, these conflicting findings may be due to methodological limitations and warrant further investigation. Ketogenic therapy has also been shown to modulate the NAD^+^/NADH (redox) ratio (*15*), improve energy metabolism, and produce clinical improvements in SZ and BD (*16*), as well as AD, mild cognitive impairment (MCI), and PD (tables S7 to S9).

In addition to the extensive body of published clinical research, numerous active clinical trials (ClinicalTrials.gov) of the ketogenic diet (*n* = 120), nicotinamide riboside (NR; *n* = 45), and nicotinamide mononucleotide (NMN; *n* = 10) reflect a rapidly growing interest in the therapeutic efficacy of redox therapies across neuropsychiatric disorders. However, the clinical efficacy of NAD^+^ precursors and ketogenic interventions, as well as their underlying molecular mechanisms, remains elusive. Furthermore, most published clinical trials lack in vivo measures of target engagement in the brain, namely NAD^+^ and NADH concentrations, as well as downstream metabolic effects on mitochondrial function. Therefore, it is unclear whether these interventions accomplish their biochemical goals or engage their targets in the brain. In addition, the efficacy of redox interventions may also depend on disease stage, patient subgroups, prescribed medications, the integrity of the glutathione (GSH) system (*17*), and intrinsic redox status (*18*, *19*). For example, research suggests that NAD^+^ supplementation may be most beneficial for individuals with the highest levels of baseline redox dysfunction (*19*). Nevertheless, the desired downstream effect of redox therapy—enhancing mitochondrial function and increasing energy supply—relies not only on higher NAD^+^ levels but also on the effective utilization of NAD^+^ at the subcellular level. Developing reliable neuroimaging methods to monitor the redox ratio and other downstream metabolic markers is critical for optimizing treatment strategies. Thus, the present manuscript will review the molecular mechanisms of redox therapies and the development of neuroimaging biomarkers. The status of published clinical trials, the dosage dependence of blood NAD^+^ levels, NAD^+^ subcellular cycling, and prospective strategies for future research will also be discussed.

## ENERGY METABOLISM, NAD, AND NEUROPSYCHIATRIC DISORDERS

Mitochondrial dysregulation and aberrant energy metabolism are increasingly recognized as critical factors in the pathophysiology of various neuropsychiatric disorders, characterized by disrupted glucose metabolism and oxidative stress (*3*, *4*, *8*, *9*). Seeing as the human brain makes up around 2% of body weight but consumes ~20% of the body’s glucose and oxygen supply, minor disruptions to energy metabolism can lead to disproportionately large changes in brain function. There is also an age-related decrease in glucose metabolism in healthy human brains, which may occur earlier and progress more rapidly in those with AD and other neurodegenerative disorders (*20*, *21*). Notably, alterations in glucose metabolism are present in people with MCI before the onset of AD (*21*), and insulin resistance is a risk factor for both AD and psychotic disorders (*22*, *23*). Emerging evidence suggests that the primary energy production mechanism in both neurodegenerative and psychotic disorders may shift from oxidative phosphorylation to glycolysis, leading to reduced adenosine triphosphate (ATP) production (*24*, *25*), increased lactate levels (*26*, *27*), a decreased redox ratio, and decreased creatine kinase (CK)/adenosine triphosphatase (ATPase) activity (*28*, *29*). ATP is the primary energy source for many biological processes. For instance, axonal integrity (*30*), neuroinflammation suppression (*31*), and waste clearance (*32*) in the brain are highly ATP-dependent. ATP is also an extracellular signaling molecule that can affect neural networks in many neuropsychiatric disorders (*33*, *34*). Thus, the brain’s ATP supply and mitochondrial function are of extraordinarily high importance in the etiology and treatment of neuropsychiatric diseases (*34*).

**Fig. 1.**
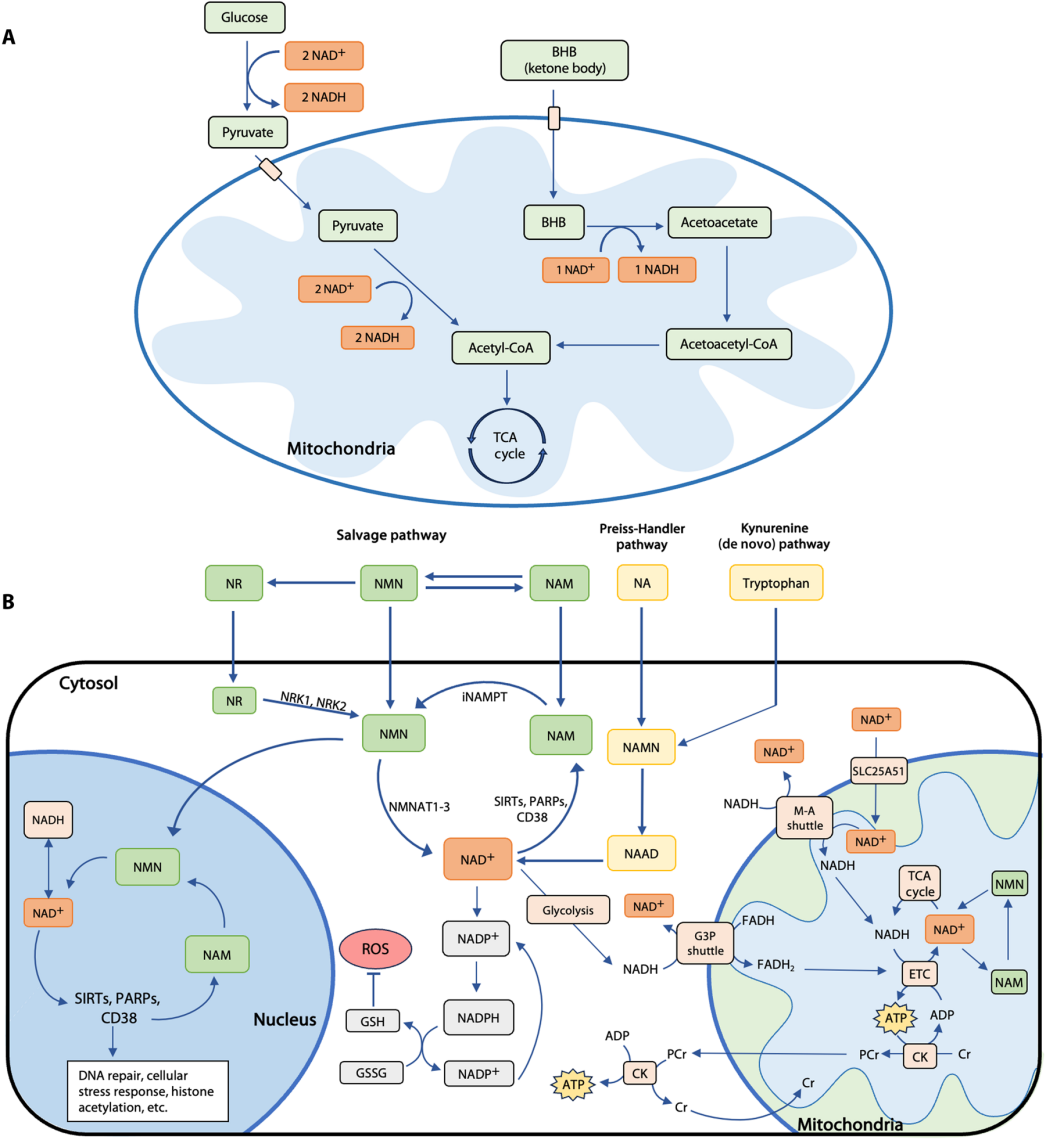
NAD cellular homeostasis. Diagram of NAD cellular homeostasis and energy metabolism. (**A**) Ketone body versus carbohydrate metabolism. β-Hydroxybutyrate (BHB) is the primary ketone body found in the brain. Glucose metabolism and ketone body metabolism are two alternative pathways for ATP production via the mitochondria. Both pathways produce acetyl–coenzyme A (acetyl-CoA), which is then used for ATP synthesis through the TCA cycle. However, the metabolism of glucose to acetyl-CoA requires four NAD^+^, while ketone metabolism only requires one NAD^+^. Therefore, ketone metabolism may decrease the need for NAD^+^ to produce energy. Given that NAD^+^ levels decline as humans age, relying on ketone metabolism could reduce competition between NAD^+^-consuming processes in the brain, while allowing for sufficient energy production. (**B**) NAD^+^ plays a key role in many cellular processes, including energy metabolism and cellular redox balance. NAD^+^-consuming enzymes [SIRTs (sirtuins), PARPs, and CD38] are critical for DNA repair, cellular stress responses, histone acetylation, and more. NAD^+^ and NADH are also important for producing GSH, the brain’s primary antioxidant. NAD^+^ levels decline as humans age, which may be a consequence of increasing CD38 and PARP levels. This decline causes other NAD^+^-consuming processes to compete over available NAD^+^, leading to increased oxidative stress, impaired energy metabolism, mitophagy, DNA damage, and eventually neuronal loss. Consumption of NAD^+^ precursor supplements may increase intracellular levels of NAD^+^. However, the use of this NAD^+^ in energy metabolic processes is dependent on transport from the cytosol to the mitochondria. Intramitochondrial NADH can be recycled into NAD^+^ through the glyceraldehyde-3-phosphate (G3P) shuttle or the malate-aspartate (M-A) shuttle. NAD^+^ can also be transported from the cytosol to the mitochondria by a mammalian mitochondrial protein, SLC25A51.

NAD^+^ and NADH play an integral role in ATP synthesis and the regulation of energy metabolism in the brain (Fig. 1) (*5*) and are essential for metabolic processes such as glycolysis, ketolysis, β-oxidation, and the tricarboxylic acid (TCA) cycle (*35*). A decrease in NAD^+^ concentration could impair multiple steps of ATP synthesis, ultimately slowing ATP production through oxidative phosphorylation and depriving the brain of sufficient energy. In addition to its role in energy metabolism, NAD^+^ serves as a key signaling molecule that regulates cell function and survival in response to environmental changes, primarily through interactions with sirtuin and poly (ADP-ribose) polymerase (PARP) signaling proteins (*11*). NAD^+^ concentrations decrease naturally as humans age (*36*, *37*), leading to competition between NAD^+^-consuming metabolic processes (*35*, *38*). This decline accelerates after the age of 45 years in humans (*35*) and is widely regarded as one of the principal contributors to the aging process, possibly due to its role in mitochondrial dysregulation and increased production of reactive oxygen species, leading to improper neuronal function (*11*). Among the downstream effects of NAD^+^ depletion, excess reactive oxygen species are strongly associated with neurodegeneration, through damage to proteins, lipids, and nucleic acids (*39*). Furthermore, animal models have demonstrated that NAD^+^ depletion can exacerbate the disease processes involved in the progression of AD (*5*). It is increasingly accepted that declining NAD^+^ levels may contribute to the progression of neurodegenerative diseases such as AD (*40*) and that energy metabolic dysfunctions arise long before the emergence of recognized AD biomarkers, such as amyloid-β plaques and neurofibrillary tangles (*41*). Disrupted redox balance and energy metabolism have also been implicated in psychotic disorders (*42*). Therefore, redox dysregulation is a promising target for therapeutic intervention and disease prevention.

## NAD^+^ SYNTHESIS PATHWAYS AND PRECURSOR SUPPLEMENTATION

NAD^+^ is synthesized through three main pathways in the cytosol and major organelles (Fig. 1), including the kynurenine (de novo) pathway, the Preiss-Handler pathway, and the salvage pathway (*19*, *43*, *44*). Through several steps, the kynurenine pathway converts tryptophan into kynurenine, which can then generate NAD^+^ (*5*). The Preiss-Handler pathway uses the precursor nicotinic acid (NA) to synthesize NAD^+^ via the deamidated form of NMN and nicotinic acid adenine dinucleotide (*5*). The salvage pathway is the primary target of interventions using oral NAD^+^ supplements (*45*). As nicotinamide (NAM) is often produced as a by-product of NAD^+^ consumption in the cell, the primary function of this pathway is to salvage NAM through conversion to NMN, which is converted back to NAD^+^. This pathway can transport extracellular NR, NMN, or NAM into the cell and convert it to NAD^+^ (*5*). Among these precursors, NR and NMN have been most extensively studied, in part because NR and NMN both have fewer side effects in humans compared to NA and NAM (*11*, *46*). In addition, NR and NMN seem to increase NAD^+^ levels more effectively than NA and NAM in rodents (*11*, *46*).

To our knowledge, a total of 40 human NAD^+^ supplementation studies have been published, including studies in AD or MCI (*n* = 5), PD (*n* = 6), psychotic disorders (*n* = 3), aging (*n* = 8), and healthy controls (*n* = 18). The limited number of publications per disorder, small and heterogeneous sample sizes, experimental constraints, and variability of available data pose substantial challenges for conducting meta-analyses across studies. Therefore, we have summarized recent clinical trials of the most researched NAD^+^ precursors—NR and NMN—below and in tables S1 to S6. Some of these findings have also been reviewed previously (*19*, *43*, *44*, *47*).

## DOSE-DEPENDENT EFFECTS OF NR AND NMN SUPPLEMENTATION ON BLOOD NAD^+^ LEVELS

**Fig. 2.**
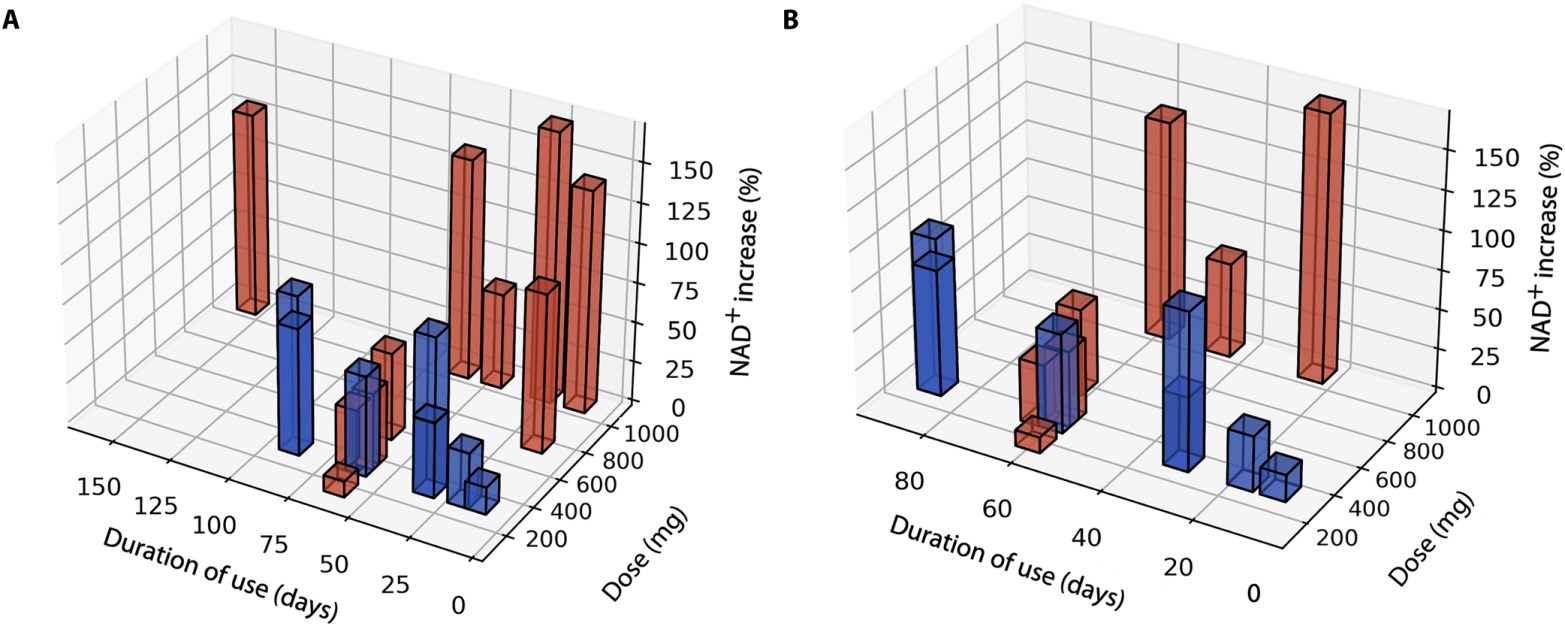
Blood NAD^+^ concentrations after supplementation. Dose-dependent effects of NR (red) and NMN (blue) supplementation on blood NAD^+^ levels in (**A**) healthy adults of all ages and (**B**) healthy adults aged 45 years and older. Each bar represents the average percent change in blood NAD^+^ concentration for a published research study. The NAD^+^ supplement duration of use and dose are indicated on the *x* and *y* axes, respectively. The data in healthy controls show that dose size, more than the duration of use, determines blood level NAD^+^ changes.

Our review indicates that NR and NMN supplementation effectively boosts blood NAD^+^ levels in healthy adults (Fig. 2). Furthermore, dose, rather than the duration of use, is the primary determinant of changes in blood NAD^+^ levels (Fig. 2). Two large studies in healthy adults have confirmed that NR supplementation results in dose-dependent increases in blood NAD^+^ levels (table S5) (*48*–*50*). One large study in older adults indicates that NMN supplementation generates a similar dose-dependent increase in blood NAD^+^ levels (table S6) (*51*). Unfortunately, NR is often studied at higher doses than NMN, making direct comparison points between supplement types rare. The single available direct comparison points suggest that NMN may be a more efficient precursor for increasing blood NAD^+^ levels than NR when taken at lower doses (Fig. 2). This efficiency may be due to NMN’s direct conversion to NAD^+^ via the salvage pathway, bypassing an additional enzymatic step required for NR (Fig. 1). Another factor influencing the efficacy of NAD^+^ supplementation may be baseline NAD^+^ concentrations. Multiple studies have found high variability in supplement-induced blood NAD^+^ levels (*50*, *51*), with lower baseline levels predicting greater increases following supplementation (*52*). Despite these findings, variations in dose, duration of use, blood NAD^+^ measurement techniques, and baseline NAD^+^ levels present challenges in standardizing comparisons across studies. These discrepancies highlight the need for further research using standardized methods to more accurately quantify the relationship between dose, duration, and NAD^+^ response.

## NAD^+^ CLINICAL TRIALS IN NEUROPSYCHIATRIC DISORDERS

Recent studies in neurodegenerative disorders have shown that NR supplementation may influence clinical, cognitive, and neurological symptoms (*53*, *54*). Of the three recent NAD^+^ precursor trials in AD or MCI, cognition either improved or remained constant (*53*–*55*). The large placebo-controlled clinical trial of NR in AD found significant improvements in cognition compared to baseline and increased concentrations of NAD^+^ and glutathione-related metabolites in serum (*54*). NR has also been shown to reduce phosphorylated tau 217 (pTau), a common biomarker of AD progression, whereas the pTau level increased in a parallel placebo group (*53*, *55*). In addition, a small trial of NR in healthy adults found decreased concentrations of another AD biomarker, amyloid-β 42, in the plasma extracellular vesicles enriched for neuronal origin of NR treatment responders (*56*).

Two clinical trials of NR supplementation have been conducted in individuals with PD. Both trials found improvements in clinical symptoms as measured by the Unified Parkinson’s Disease Rating Scale (UPDRS) (*57*, *58*). Furthermore, one trial in PD found an increase in brain total NAD (the sum of NAD^+^ and NADH) concentration observed by in vivo ^31^P magnetic resonance spectroscopy (^31^P-MRS) after NR supplementation (*58*). Notably, improvement in UPDRS scores was significantly correlated with NR-induced increases in cerebral total NAD concentrations (*58*). However, most published clinical trials in individuals with neurodegenerative disorders, particularly in PD, have treatment groups with small sample sizes—typically 15 or fewer.

Clinical trials of NMN have primarily focused on safety assessment and aging-related outcomes such as muscle function and mobility. To date, clinical studies have consistently found that NMN is well tolerated in healthy adults at doses up to 2000 mg. These studies have found some positive health effects, including enhanced insulin sensitivity and signaling (*59*) and reduced fatigue (*60*). However, NMN supplementation has had mixed effects on mobility and muscle function in older adults (*51*, *60*–*63*). To our knowledge, no clinical trials of NMN supplementation in neurodegenerative or psychiatric disorders have been published, and trials in aging adults have not measured cognition.

Despite emerging evidence that various psychiatric disorders are associated with redox dysregulation and impaired brain energy metabolism (*16*, *24*), clinical trials of NAD^+^ supplementation in this population are limited. Only case studies, rather than clinical trials, of NAD^+^ supplements in psychotic disorders and BD have been published (table S3). Although existing clinical trials of NR supplementation in neurodegenerative disorders are promising, the clinical utility of these supplements in psychiatric disorders is still unclear and warrants further investigation.

## OTHER FINDINGS, NAD^+^ SUBCELLULAR CYCLING, AND PERSPECTIVES FOR THE FUTURE

Existing literature has focused on increasing NAD^+^ concentrations and rebalancing the redox ratio. However, the desired downstream effect of redox therapy—enhanced mitochondrial function and increased energy supply—relies not only on higher NAD^+^ levels but also on the effective utilization of NAD^+^ at the subcellular level. While NR and NMN can offer a strong biosynthetic effect without significant adverse effects, increased concentrations may not enter or be used efficiently by the mitochondria. For example, NAD^+^ production may occur more slowly than NR breakdown, as blood NAD^+^ levels increase and plateau after supplementation, while blood NR levels peak and decline (*50*). NR has a short half-life, so multiple doses of NR per day may allow for more consistent NR production (*50*). The cellular/subcellular cycling and kinetics of NR, NMN, and NAD^+^ are not well understood and merit further investigation.

Recent studies suggest that both cellular and mitochondrial NAD^+^ transporters may impose additional constraints on the therapeutic efficacy of NAD^+^ precursor supplements (*64*–*66*). For example, organelle-level utilization of NAD^+^ is rate limited by nicotinamide riboside kinases (NRKs), the enzyme responsible for the phosphorylation of NR into NMN (Fig. 1) (*66*). To address the limitations imposed by NRKs, researchers have recently identified a reduced form of NR, NRH, which is more potent and faster-acting than NR (*66*). Unlike NR, NRH uses an NRK-independent pathway, potentially eliminating the upper limit on the rate and magnitude of NAD^+^ synthesis (*66*). This hypothesis warrants validation in human studies.

In prior work, NR was considered superior to NMN because it was believed to enter cells through a highly complex pathway (*11*). However, researchers have recently identified a novel mammalian NMN transporter, the Slc12a8 protein, that directly facilitates NMN transport (*65*). The *Slc12a8* gene is typically up-regulated in response to declining NAD^+^ concentrations (*65*), indicating its role in compensating for a reduced redox ratio. *Slc12a8* knockout mice have impaired NMN uptake and rates of NAD^+^ synthesis (*65*). Furthermore, older mice tended to up-regulate *Slc12a8* significantly more than younger ones, perhaps in an attempt to combat decreasing NAD^+^ levels (*65*). Up-regulating this gene or supplementing the Slc12a8 protein in combination with NMN has the potential to compensate for NMN’s slow uptake speed.

Research has also revealed that the SLC25A51 protein and its paralog, SLC25A52, act as transporters that carry NAD^+^ from the cytoplasm into mitochondria (*64*). Experimental evidence suggests that the loss of SLC25A51 protein reduces mitochondrial NAD^+^ concentrations and impairs mitochondrial respiration without affecting whole-cell NAD^+^ levels, but increasing SLC25A51 availability boosts mitochondrial NAD^+^ content (*64*). This might explain the modest clinical effects observed in current clinical trials of oral NAD^+^ precursor supplementation. Although oral supplements effectively increased blood NAD^+^ levels, the elevated NAD^+^ may not enter mitochondria at a rate proportional to the increase in blood concentrations, or at all, because there are not enough transporters available. Up-regulation of these genes or supplementation of the protein may enhance the delivery of elevated blood NAD^+^ concentrations into the brain’s mitochondria. Investigating the function of these transport proteins and NAD cellular/subcellular cycling is essential for improving the efficacy of NAD-boosting therapies.

In addition to its role in energy metabolism, NAD^+^ is a key modulator of various cellular signaling and survival pathways (*29*, *47*, *67*), including cellular metabolism, DNA repair, immune responses, and circadian rhythm regulation (*5*, *68*–*71*). Thus, it is crucial to understand that NAD^+^ supplements may have widespread effects on the brain. In future clinical research, it will be important to consider both the potential therapeutic effects and consequences of altering these additional cellular pathways. However, this discussion is beyond the scope of the current manuscript.

## KETOGENIC THERAPY

Ketogenic therapies, including the ketogenic diet and ketosis-inducing supplements, modulate energy metabolism (*15*) and have been used for more than 100 years to treat illnesses characterized by metabolic stress, such as refractory epilepsy (*72*). Ketogenic therapy has received increasing attention as a potential intervention for neurodegeneration and neuroinflammation (*73*, *74*). This treatment can cause the brain to rely more heavily on ketones, an alternative energy source to glucose (Fig. 1). Therefore, people with disorders characterized by disrupted glucose metabolism, such as AD, may benefit from ketogenic interventions.

Across clinical trials, both ketogenic supplements and the ketogenic diet have effectively increased both blood and brain ketone concentrations (tables S7 to S9) (*75*–*79*). In AD and MCI, ketogenic therapies have produced broad improvements in cognitive function (*76*–*78*, *80*) that are associated with increases in brain and blood ketone concentrations (table S7) (*76*–*78*). Notably, Kumar *et al.* found reduced plasma levels of amyloid-β 1–42, p181 tau, and neurofilament light chain in small extracellular vesicles from participants with MCI after a 6-week modified Mediterranean ketogenic diet (*81*). However, another group found no significant change in plasma levels of these AD biomarkers following 6 months of a medium-chain triglyceride drink (*82*). The ketogenic diet has also yielded encouraging results in patients with PD, with all four recent published trials reporting improvements in clinical symptoms, including vocal impairment (table S8) (*83*–*86*). In addition, significant diet-induced improvements of nonmotor PD symptoms, measured by part 1 of the UPDRS, have been replicated in three clinical trials (*83*, *84*, *86*). However, the ketogenic diet has not produced significant reductions in motor symptoms of PD (*83*, *84*, *86*).

A recent review suggests that the ketogenic diet may be a transdiagnostic treatment that can improve symptoms in various neuropsychiatric disorders, including psychotic disorders and BD (*74*). Two recent open-label studies in SZ and BD have provided evidence supporting the potential utility of the ketogenic diet to improve psychiatric symptoms (*87*, *88*). One study found a significant correlation between serum ketone concentrations and self-reported symptoms of mood, energy, impulsivity, and anxiety (*88*). A retrospective case series and case reports have also reported beneficial clinical effects in psychotic disorders (table S9) (*74*, *89*). In contrast to PD and AD, ketogenic therapy trials in psychiatric disorders have not included high-carbohydrate diet comparison groups.

Notably, beyond shifting the brain’s primary energy source from glucose to ketones, there is an NAD^+^-sparing effect exhibited in the switch from glycolysis to ketolysis (*15*). Animal studies have consistently shown that nutritional ketosis can modulate the redox ratio (*16*). Furthermore, a recent study in healthy adults showed that nutritional ketosis increased brain NAD^+^ concentrations and decreased NADH concentrations using ^31^P-MRS (*15*). Additional biological connections between NAD metabolism and ketogenic therapy may exist, as NMN supplementation has been shown to increase ketone production and down-regulate mitochondrial oxidative phosphorylation and TCA cycle components in the mouse brain (*90*). However, the extent to which the clinical effects of ketogenic therapy are related to this NAD^+^-sparing effect remains unclear and should be investigated further (*16*).

## IN VIVO BIOMARKER DEVELOPMENT FOR REDOX THERAPY

Undoubtedly, the development of effective redox therapies relies on our ability to noninvasively measure the effect of interventions on target engagement. However, few published clinical trials have measured NAD^+^ concentrations or the redox ratio at critical tissue sites following NAD^+^ supplementation or ketogenic therapies. A similar sentiment is offered by Fletcher and Lavery (*91*), who emphasize a lack of tissue and organelle specificity, technical limitations of NAD^+^ quantification, and the need for more robust biomarkers to monitor NAD^+^ cycling in health and disease as major obstacles in clinical research.

### In vivo redox ratio quantification

Although NAD metabolism is increasingly recognized as an integral factor of neuropsychiatric disease, measuring the NAD^+^/NADH redox state is exceptionally challenging. Despite the fact that ex vivo blood assays and autofluorescence methods have been developed, sample preparation remains difficult because of the highly sensitive nature of NAD^+^ and NADH (*91*). In addition, these methods do not provide a direct measure of metabolite levels in the living human brain. Thus, alternative approaches are needed to accurately and reliably quantify these metabolites in vivo.

**Fig. 3.**
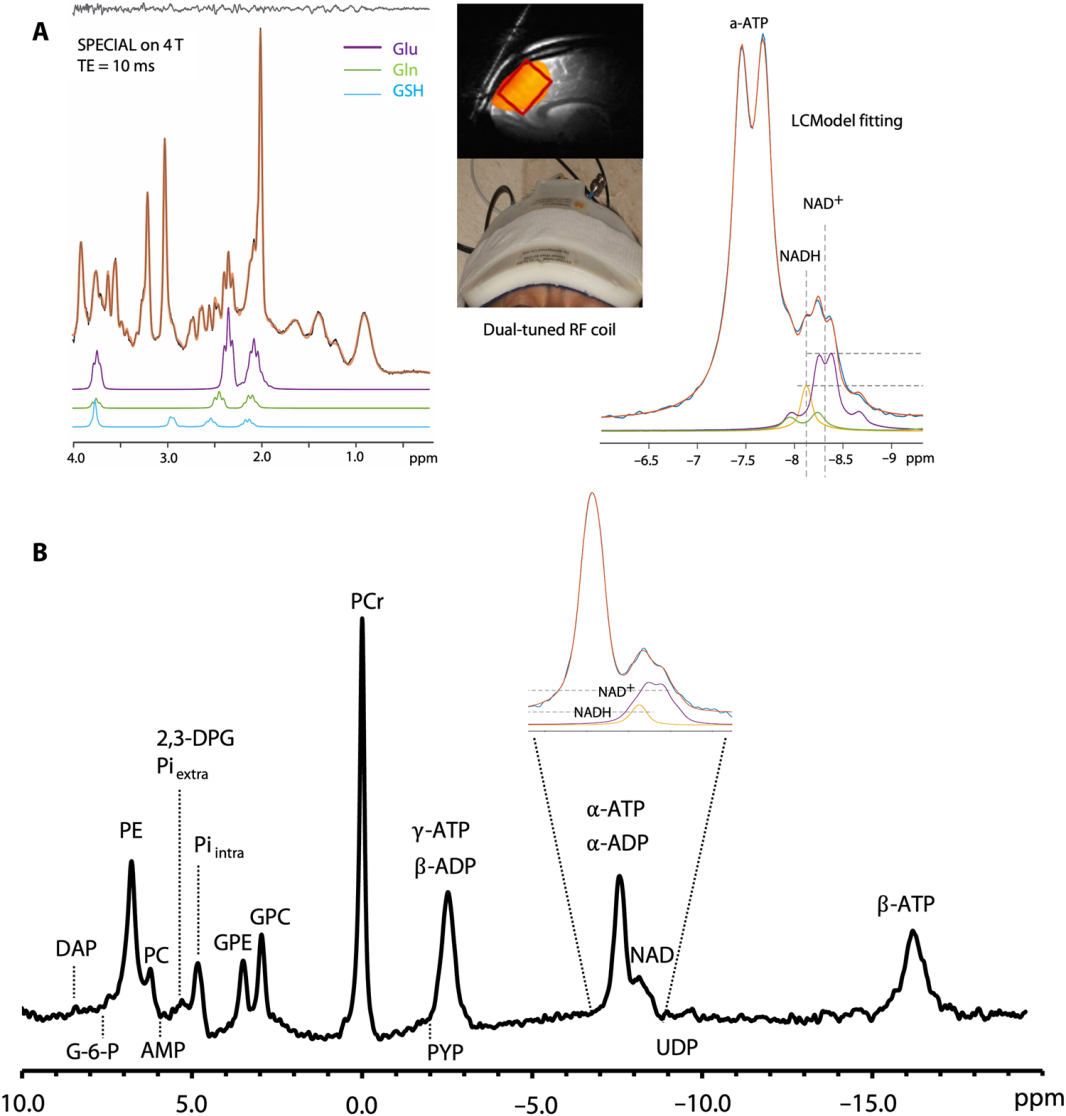
NAD measurement using MRS. (**A**) Schematic plots of ^1^H-MRS (left) and ^31^P-MRS (right). Anatomic imaging and the radio frequency (RF) coil used (middle) at a 4T scanner are shown. The yellow color and red box indicate the sensitivity region of the ^31^P-MRS RF coil and the ^1^H-MRS voxel replacement [TR (repetition time)/TE (echo time) = 3000/10 ms], respectively. The partial spectroscopy curve fitting and the signals of metabolites are also illustrated. With an ultrahigh-field magnetic resonance imaging scanner and dedicated experimental design (e.g., dual-tuned RF surface coil and ^31^P-MRS with ^1^H decoupling at 4T), both ^1^H-MRS and ^31^P-MRS can be obtained in the same experimental session. This allows quantification of Glu, glutamine (Gln), GSH, myo-inositol, NAD^+^/NADH, and CK/ATPase activity. (**B**) In vivo ^31^P-MRS from the occipital lobe obtained from a 7T scanner. P_i_, PCr, and γ-ATP resonances are chemically coupled through the chemical reactions of CK and ATPase, whose activities can be assessed by ^31^P-MRS with magnetization transfer approaches. Phosphoethanolamine (PE), phosphocholine (PC), glycerophosphoethanolamine (GPE), and glycerophosphocholine (GPC) are related to cell membrane lipids. The tissue pH value and magnesium (Mg^2+^) concentration can be determined from the chemical shift difference of P_i_ and β-ATP relative to PCr. ppm, parts per million; DAP, dihydroxyacetone phosphate; G-6-P, glucose-6-phosphate; AMP, adenosine monophosphate; ADP, adenosine diphosphate; PYP, phosphoenolpyruvate; UDP, uridine diphosphate.

MRS provides a noninvasive means of measuring metabolites in the human brain. The most viable option is ^31^P-MRS, although this type of measurement is often challenging because of low NAD^+^ and NADH concentrations (<1 mM) and overlapping resonances. This method typically requires ultrahigh-field magnetic resonance scanners (4 or 7T), allowing separate quantification of NAD^+^ and NADH concentrations (Fig. 3) (*36*, *92*, *93*). ^31^P-MRS has been successfully used to measure NAD^+^ levels and the redox ratio in animal models (*94*) and humans with various neurological and psychiatric disorders (*15*, *29*, *36*, *95*, *96*). Our group has used this method to quantify reductions in the redox ratio in patients with chronic and first-episode psychosis (*29*). Moreover, our findings show an expected age-related decline in NAD^+^ concentrations, consistent with other reports (*35*, *36*). Downfield ^1^H-MRS has also been successfully used to measure NAD^+^ (*97*–*99*) and the effect of NR on brain NAD^+^ concentrations at a 7T scanner (*100*). Although ^1^H-MRS cannot separately quantify NADH concentrations, it is more widely accessible and may serve as a useful alternative to ^31^P-MRS for assessing the redox therapy treatment response.

To our knowledge, only two trials have used MRS to measure the effect of NAD^+^ precursor supplementation on brain NAD concentrations. One trial found an increase in total NAD after NR supplementation in patients with PD using ^31^P-MRS at a 3T scanner (*58*). Another study reported increased NAD^+^ concentrations in healthy adults using ^1^H-MRS at 7T after NR supplementation (*100*). MRS has also been used to quantify the effects of nutritional ketosis; a single trial successfully measured an increase in redox ratio using ^31^P-MRS at 7T during nutritional ketosis in healthy participants (*15*). These examples demonstrate the feasibility of measuring redox therapy target engagement in the brain using both ^31^P-MRS and ^1^H-MRS. As previously discussed, individuals with normal baseline NAD^+^ levels may not benefit as substantially from NAD^+^ supplementation compared to those with low brain NAD^+^ levels (*19*). Therefore, NAD^+^ quantification before intervention initiation may be a valuable approach for guiding personalized interventions in disorders characterized by redox dysregulation.

### In vivo biomarkers for mitochondrial function and oxidative stress

In addition to measuring NAD^+^ and NADH concentrations, ^31^P-MRS can assess downstream effects of redox therapy on mitochondrial function. Metabolites such as phosphocreatine (PCr), ATP, and inorganic phosphate (P_i_) are biochemically coupled via CK and ATPase enzyme activity; these chemical reactions are essential for regulating ATP metabolism. Researchers have typically used the ratios between PCr, ATP, and P_i_ to assess ATP metabolism in the brain (Fig. 3). However, most studies have ignored chemical exchange and T1 relaxation effects, leading to inaccurate measurements (*101*, *102*). Accordingly, investigators have developed cutting-edge techniques to dynamically quantify ATPase and CK reaction rates in the brain (*101*–*103*). Using these techniques, our group found that CK activity is impaired in patients with SZ despite normal ATP and PCr levels (*9*). These dynamic measurements are preferable to static ones because ATP and PCr levels may remain constant through compensation from other sources, even if CK enzymatic activity is impaired (*9*, *104*). Therefore, the dynamic assessment of CK and ATPase is a valuable tool for assessing the downstream effects of redox therapy.

**Fig. 4.**
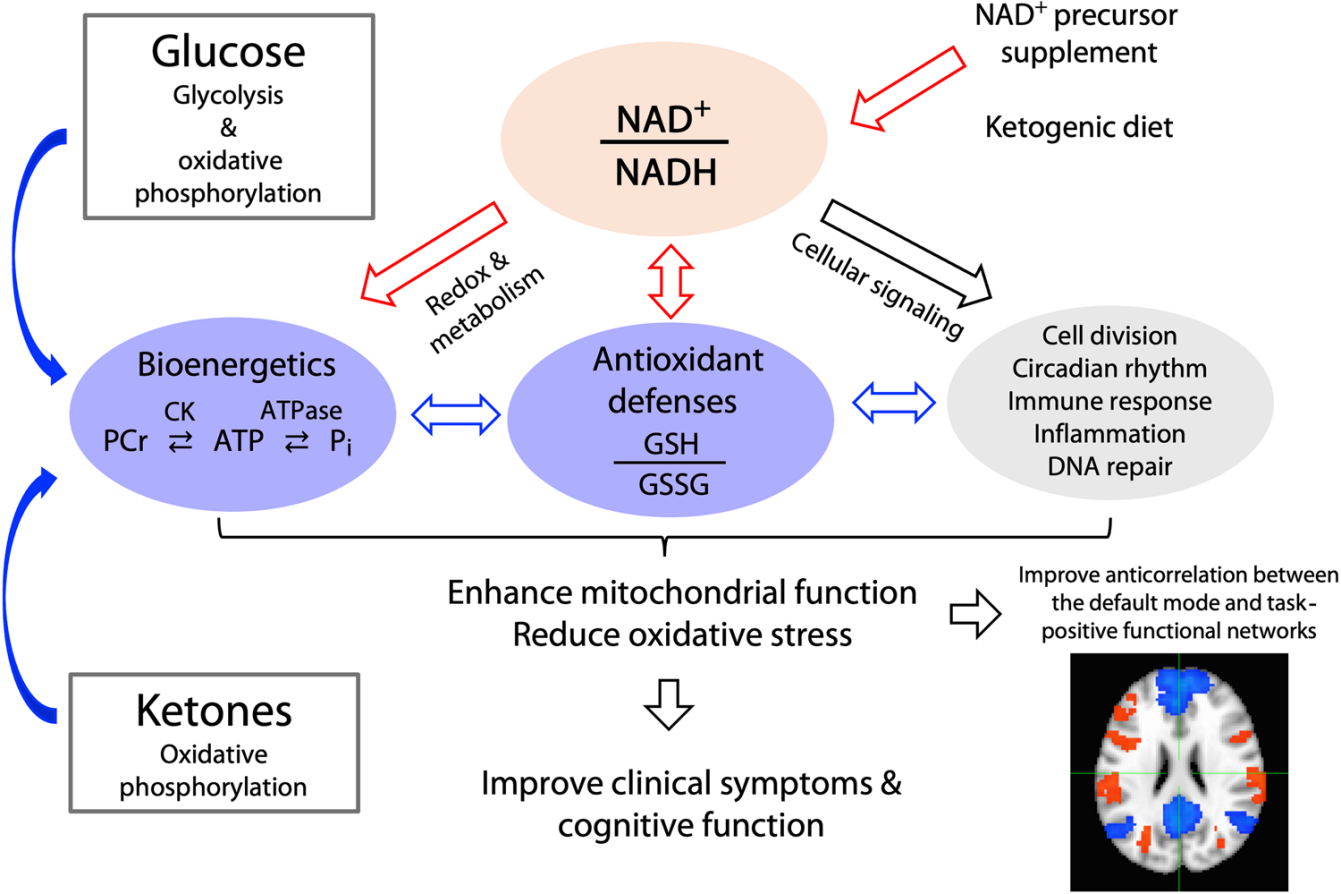
Effects of NAD^+^ supplementation and ketogenic therapy. The target of redox therapy is redox balance (NAD^+^/NADH ratio) and downstream effects on mitochondrial function (CK activity, ATPase activity, and GSH levels). Ketones can serve as an alternative brain energy source to glucose and can also influence the redox ratio. Through balancing the redox ratio and improving mitochondrial function, we may be able to reduce oxidative stress and facilitate other NAD^+^-dependent processes. In addition, disrupted functional brain networks may be improved with redox therapies. Patients taking NAD^+^ or ketogenic therapies have reported improvements in clinical symptoms and cognitive function. GSSG, oxidized glutathione.

In addition, ^31^P-MRS provides a measure of intracellular pH (Fig. 3), and ^1^H-MRS can quantify concentrations of GSH, lactate, glutamate (Glu), and γ-aminobutyric acid (GABA). In conjunction with decreased intracellular pH, lactate buildup may reflect reduced electron transport chain activity and tissue acidification. Thus, multinuclear MRS can be used to determine whether alterations in brain redox ratio influence energy metabolism and antioxidant defenses, such as GSH. Advanced ultrahigh-field multinuclear MRS offers a unique opportunity to meet this challenge as it provides substantially increased sensitivity and spectral resolution (*105*). Thus, in vivo measures of the redox ratio, mitochondrial function, and the brain’s antioxidant defenses can be acquired within a single experimental session (Fig. 3). Collectively, these technological advancements could herald previously unexplored approaches to developing and validating treatment strategies for neuropsychiatric disorders (Fig. 4).

### Linking brain metabolism and function

Neuropsychiatric disorders are often characterized by disrupted brain circuitry, which can be measured using resting-state functional magnetic resonance imaging. Bioenergetic processes are crucial for maintaining normal neurotransmission, synaptic connectivity, and neuronal signaling because of their high energy demand (*28*, *29*, *106*), particularly for excitatory glutamatergic cells (*107*) and fast-spiking inhibitory interneurons (*108*). Maintenance of functional networks through coordinated neural activity requires even more energy (*106*). The relationship between bioenergetic processes and functional connectivity has been documented in our recent research. We found that the CK reaction rate and GABA/Glu concentrations in the medial prefrontal cortex were associated with functional connectivity in healthy controls but were compromised in those with psychotic disorders (*28*, *109*, *110*). These findings indicate that dysregulated energy metabolism and neurotransmission may disrupt large-scale neuronal communication in psychotic disorders (*28*, *29*). Therefore, addressing disruptions to brain energy metabolism could improve neural circuit connectivity in neuropsychiatric disorders (*28*, *111*). Furthermore, measurements of functional connectivity could indicate whether redox therapies have successfully engaged with downstream targets. The use of multimodal neuroimaging biomarkers in combination with clinical data has the potential to facilitate personalized treatment approaches for a variety of neuropsychiatric conditions.

## CONCLUSIONS

New therapies are urgently needed for various neurodegenerative and psychiatric conditions. Redox therapies, such as NAD^+^ supplementation and the ketogenic diet, may address the dysregulated energy metabolism and redox imbalance observed under the neuropsychiatric conditions. NAD^+^ supplements have been proven to increase blood NAD^+^ levels effectively and have produced some promising improvements in cognitive function and clinical symptoms in neurodegenerative disorders. Ketogenic therapies have demonstrated similar clinical effects in neurodegenerative conditions, as well as improvements in psychiatric symptoms in psychotic disorders and BD. However, the clinical efficacy of redox therapies, particularly in psychiatric disorders, requires additional validation through large, placebo-controlled trials. Furthermore, the biological mechanisms underlying clinical improvements remain unclear, and most clinical studies have not used neuroimaging to verify target engagement or downstream effects on mitochondrial function in the human brain. This gap underscores the need for advanced neuroimaging techniques to elucidate molecular mechanisms in vivo. These neuroimaging techniques may also be used to create personalized interventions by measuring baseline levels of energy metabolism and redox dysregulation. Future studies can build on a broad foundation of existing research by exploring novel molecular mechanisms and their therapeutic applications, such as transporter molecules and modified precursors, while incorporating advanced neuroimaging techniques. Together, these approaches can enable the measurement of the effects of metabolic interventions at the molecular level in human subjects, paving the way for personalized, mechanism-driven treatments.

## Supplementary Materials

### This PDF file includes:

Supplementary Text Tables S1 to S13 References
