# Supplementary material for: Redox therapy for neuropsychiatric disorders: Molecular mechanisms and biomarker development
Source: Sci Adv. 2026 Feb 18;12(8):eaea9014. doi: 10.1126/sciadv.aea9014 (PMC12915611; doi:10.1126/sciadv.aea9014)
Supplement: Supplementary file 1 — Supplementary Text Tables S1 to S13 References [file sciadv.aea9014_sm.pdf]

Science Advances
AAAS

# Supplementary Materials for

## Redox therapy for neuropsychiatric disorders: Molecular mechanisms and biomarker development

Kyle W. Cuklanz *et al.*

Corresponding author: Fei Du, fdu@mclean.harvard.edu

*Sci. Adv.* **12**, eaea9014 (2026)
DOI: 10.1126/sciadv.aea9014

**This PDF file includes:**

Supplementary Text
Tables S1 to S13
References

## Supplementary Text

Materials and Methods: Systematic Review Article Search and Selection Process

The search criteria listed below were used to identify published journal articles on the PubMed database until March 1, 2025. Two qualified individuals independently implemented a literature search, which included a preliminary examination of titles and abstracts, followed by a review of full-text articles. Eighty-seven articles met search criteria 1, 211 articles met search criteria 2, 786 articles met search criteria 3, and 254 articles met search criteria 4. 1320 articles were screened based on title and abstract. Of these studies, 152 underwent a full-text screening process, and 72 studies were finally included. Additionally, we manually reviewed all references from the selected articles and previously published review papers on the same topics covered in this review and extracted relevant additional articles. Articles identified were screened as abstracts, and after the exclusion of those that did not meet our inclusion criteria, the full texts of the remaining articles were assessed for eligibility, and decisions were made regarding their inclusion in this systematic review.

The listed search criteria were used to identify published journal articles on PubMed.

1. NMN search criteria: ("nicotinamide mononucleotide"[MeSH Terms]) AND ((intervention) OR (dose) OR (placebo) OR (cognit*) OR (psych*) OR (neuro*) OR ("Schizophrenia Spectrum and Other Psychotic Disorders" [MeSH Terms]) OR ("Bipolar and Related Disorders" [MeSH Terms]) OR ("Alzheimer Disease" [MeSH Terms]) OR ("Parkinson Disease" [MeSH Terms]) OR (mild cognitive impairment)) NOT ("Review"[Publication Type]) NOT ("rodentia" [MeSH])– 87 screened from title and abstract (PubMed), 29 underwent full-text screening (PubMed), 18 were identified as eligible (total).

2. NR search criteria: (nicotinamide riboside) AND ((intervention) OR (dose) OR (placebo) OR (cognit*) OR (psych*) OR (neuro*) OR ("Schizophrenia Spectrum and Other Psychotic Disorders" [MeSH Terms]) OR ("Bipolar and Related Disorders" [MeSH Terms]) OR ("Alzheimer Disease" [MeSH Terms]) OR ("Parkinson Disease" [MeSH Terms]) OR (mild cognitive impairment)) NOT ("Review"[Publication Type]) NOT ("rodentia" [MeSH]) – 211 screened from title and abstract, 27 underwent full-text screening (PubMed), 15 were identified as eligible (total).

3. Other NAD supplement search criteria: ((NADH) OR (niacin) OR (NAD)) AND (("Schizophrenia Spectrum and Other Psychotic Disorders" [MeSH Terms]) OR ("Bipolar and Related Disorders" [MeSH Terms]) OR ("Alzheimer Disease" [MeSH Terms]) OR ("Parkinson Disease" [MeSH Terms]) OR (mild cognitive impairment)) NOT ("Review"[Publication Type]) NOT ("rodentia" [MeSH]) – 768 screened from title and abstract, 48 underwent full text screening, 9 were included

4. Ketogenic therapy search criteria: ((medium chain triglyceride) OR (MCT) OR (kMCT) OR (beta-hydroxybutyrate) OR ("3-Hydroxybutyric Acid"[MeSH Terms]) OR ("Diet, Carbohydrate-Restricted" [MeSH Terms])) AND (("Schizophrenia Spectrum and Other Psychotic Disorders" [MeSH Terms]) OR ("Bipolar and Related Disorders" [MeSH Terms]) OR ("Alzheimer Disease" [MeSH Terms]) OR ("Parkinson Disease" [MeSH

Terms]) OR (mild cognitive impairment)) NOT ("Review" [Publication Type]) NOT ("rodentia" [MeSH Terms]) – 254 screened from title and abstract, 48 underwent full-text screening, 30 were identified as eligible (total)

Inclusion Criteria for NAD Supplements:
The inclusion criteria differed for two groups of interest (healthy adults vs. adults with neuropsychiatric disorders). Articles related to healthy adults included the following: (1) adults were not diagnosed with a neurological disorder or psychiatric disorder, (2) participants received either NR or NMN supplementation, and (3) blood or brain NAD metabolites were measured. Articles related to adults with a neuropsychiatric disorder included the following: (1) adults were diagnosed with a psychotic disorder, bipolar disorder, mild cognitive impairment, Alzheimer's Disease, Parkinson's Disease, or were described as "aging," (2) received NR, NMN, NA, NAD, or NADH supplementation, and (3) measure brain or blood NAD metabolites, cognition, or impact on symptoms of a neuropsychiatric disorder.

Inclusion Criteria for Ketogenic Therapies:
Articles were included in the systematic review if they met the following criteria: (1) adults were diagnosed with a psychotic disorder, bipolar disorder, mild cognitive impairment, Alzheimer's Disease, or Parkinson's Disease, (2) received the ketogenic diet, a high-fat, low-carbohydrate diet, or a medium chain triglyceride supplement, and (3) measured brain or blood ketone body concentrations, NAD metabolites, cognition, or impact on symptoms of a neuropsychiatric disorder.

Exclusion Criteria:
Articles were excluded if they were review papers or protocols.

Materials Methods: Systematic Review Data Collection and Synthesis
Two individuals independently collected data from each identified article. The variables collected for all articles include: author, year of publication, sample size, study population group, presence of placebo group (and sample size, if applicable), type of intervention received, dose of intervention, length of intervention, and primary outcome measures. The following variables were collected from articles, if reported: change in blood NAD+, total NAD, and/or NAD+/NADH concentrations compared to baseline, change in blood NAD+, total NAD, and/or NAD+/NADH concentrations compared to a placebo group, change in brain or CSF NAD metabolite concentrations, change in brain or CSF ketone body concentrations, and change in cognition. Additional key findings from each article may be included. A p-value is reported for each outcome measure. Percent change in blood NAD+, total NAD, or NAD+/NADH was collected, if reported. Mean age and age range were collected for articles reporting blood NAD+ levels in healthy controls and patients.

Results regarding outcomes of interest from all 72 studies are reported in the tables below. We included an additional table summarizing the results of all articles measuring blood NAD levels in healthy controls. Data from articles that measured whole blood NAD+ concentrations were included in Figure 2. We excluded data from articles measuring total NAD or NAD+/NADH ratio in Figure 2. Additionally, we excluded data from articles measuring serum or PBMC NAD+ concentrations, rather than whole blood, from Figure 2. Additionally, one study was

excluded from the NMN graph due to baseline NAD+ whole blood concentrations that were vastly inconsistent with previous reports.

We opted not to apply the PRISMA reporting guidelines to this review due to its multidisciplinary scope. The manuscript integrates discussion of the molecular mechanisms underlying NAD-related bioenergetic deficits, recent advances in the development of neuroimaging biomarkers, and current and emerging NAD-targeted therapies, with an emphasis on personalized, mechanism-driven approaches. A central focus is identifying key challenges that hinder the translation of biomedical research into effective clinical treatments, along with strategies to overcome these barriers.

A systematic literature search was conducted for one section of the manuscript (clinical trials of NAD+ supplements and ketogenic therapies) to summarize the status of redox therapy. However, the limited number of publications per disorder, small and heterogeneous sample sizes, methodological constraints, and variability in available clinical trial data, particularly in biomarker studies, posed significant challenges for data synthesis. Therefore, we combined the systematic review of clinical trials with additional topics to provide a comprehensive perspective on the field's current progress and remaining challenges.

List of Abbreviations

| Abbreviation | Definition |
|---|---|
| AB42 | Amyloid-beta-42 |
| AD | Alzheimer's Disease |
| Aging | Cognitive aging |
| ALS | Amyotrophic lateral sclerosis |
| ATP | Adenosine triphosphate |
| BD | Bipolar disorder |
| CK | Creatine kinase |
| GABA | Gamma-aminobutyric acid |
| Glu | Glutamate |
| GSH | Glutathione |
| MCI | Mild cognitive impairment |
| MRS | Magnetic resonance spectroscopy |
| NA | Nicotinic acid |
| NAAD | Nicotinamide acid adenine dinucleotide |
| NAD+ | Nicotinamide adenine dinucleotide (oxidizing form) |
| NADH | Nicotinamide adenine dinucleotide (reducing form) |
| NAM | Nicotinamide |
| NMN | Nicotinamide mononucleotide |
| NR | Nicotinamide riboside |
| NRK | Nicotinamide riboside kinase |
| PCr | Phosphocreatine |
| PD | Parkinson's Disease |
| Pi | Inorganic phosphate |

| | |
|---|---|
| pTau | Phosphorylated Tau 217 |
| Redox | NAD+/NADH |
| SZ | Schizophrenia |
| TCA cycle | Tricarboxylic acid cycle |
| UPDRS | Unified Parkinson’s Disease Rating Scale |
| UHF | Ultra-high field |

**Table S1.**

| *NAD Supplements* | | | | |
|---|---|---|---|---|
| **AD and MCI** | | | | |
| **Author** | **Intervention (type, dose, length)** | **Population (condition, n-enrolled subjects)** | **Primary Outcome(s)** | **Results (brief description)** |
| **Wu et al., 2025** *(55)* | ***NR,*** 1000 mg, 8 weeks | ***MCI*** (n=41) | Plasma phosphorylated tau 217 (pTau) concentrations and cognition | pTau was reduced by 7% during NR phase and increased by 18% during placebo phase (on average across both sequence groups) (p=0.02). No change in cognition. |
| **Orr et al. 2024** *(53)* | ***NR,*** 1000 mg per day, 10 weeks | ***MCI;*** treatment (n=10) vs placebo (n=10) | Post-treatment cognition, cerebral blood flow, blood NAD+ levels | Increased blood NAD+ levels by 160% (p<0.001); Decreased cerebral blood flow in the DMN (p=0.013); Cognitive effects were not statistically significant. |
| **Yulug et al., 2023** *(54)* | ***NR,*** 1000 mg**,** 12.35 g L-serine, 2.55 g N-acetyl-L-cysteine, and 3.73 g L-carnitine tartrate. 1 dose/day from 1-28 days, 2 doses/day from 29-84 days | ***AD;*** treatment (n=47), placebo (n=22) | Cognitive function and daily living activity scores (primary), plasma metabolome and proteome analysis (secondary) | Improved cognition in NR group (p<0.001), slight improvement in cognition by 14% in placebo group (p=0.001); Improvements in the plasma levels of metabolites associated with NAD+ and glutathione metabolism after CMA treatment (p<0.05). |
| **Demarin et al., 2004** (*112)* | ***NADH***, 10 mg/day, 6 months | ***AD*** (n=26); (13 ***NADH***, 13 placebo) | Cognition | Improved verbal fluency (p=0.019) and visual constructional ability (p=0.038) in NADH group. |
| **Rainer et al., 2000** *(113)* | ***NADH***, 1000 mg/day, 10 weeks | ***Mild to Moderate Dementia*** (n=19) | Cognition | No change in cognition. |

**Published trials of NAD supplements in AD and MCI.** Published studies of NAD supplements in AD and MCI (n=5) have measured the effect of NR or NADH supplementation on cognitive function, blood NAD+ concentration, cerebral blood flow, plasma metabolome and proteome, and plasma pTau concentrations. Notably, every published trial found that cognition improved or remained constant, and one trial found reduced plasma pTau levels.

**Table S2.**

| *NAD Supplements* | | | | |
|---|---|---|---|---|
| **Parkinson's Disease** | | | | |
| **Author** | **Intervention (type, dose, length)** | **Population (condition, n-enrolled subjects)** | **Primary Outcome(s)** | **Results (brief description)** |
| **Berven et al., 2023** *(57)* | 3000 mg ***NR***/day for four weeks | ***Parkinson's Disease;*** treatment (n=10) vs placebo (n=10) | Safety, tolerability, change in whole blood and urine NAD metabolome, change in clinical severity of PD | Improvement in MDS-UPDRS (p=0.02); 256% increase in blood NAD+ (p<0.001). |
| **Brakedal et al., 2022** *(58)* | 1000 mg ***NR***/day for 30 days | ***Parkinson's disease;*** treatment (n=15) vs placebo (n=15) | Brain NAD levels (31P-MRS), cerebral metabolism (FDG-PET), CSF metabolomics | Increase in brain total NAD levels (p=0.016) compared to baseline; Decreased glucose uptake in basal ganglia and neocortical areas; Increased transcription of mitochondrial respiration genes in serum (p<0.01) and decrease in inflammatory cytokines (p<0.05); improvement of MDS-UPDRS scores associated with NR-induced increase in cerebral NAD (p=0.0017). |
| **Kuhn et al., 1996** *(114)* | ***NADH*** | ***Parkinson's disease*** (n=15) | MDS-UPDRS, plasma levodopa levels | Significant MDS-UPDRS improvement (p=0.025); Increased bioavailability of plasma levodopa (p<0.05). |
| **Dizdar et al., 1994** *(115)* | 25 mg IV ***NADH***/day, for 4 days, followed by 25 mg IM ***NADH*** at 2 weeks and 4 weeks after last IV treatment | ***Parkinson's disease;*** treatment (n=5) vs placebo (n=4) | MDS-UPDRS | Trend towards clinical improvement (p>0.05). |
| **Birkmayer et al., 1993** *(116)* | 12.5 mg ***NADH*** by IV or 5 mg NADH orally by capsules | ***Parkinson's disease;*** IV (n=415) vs oral (n=470) | Clinical disability according to the Birkmayer and Neumayer scale | Clinical improvement in 80% of patients. |
| **Birkmayer & Birkmayer, 1989** *(117)* | 25-50 mg ***NADH***/day, IV or IM | ***Parkinson's disease*** (n=34) | Clinical disability according to the Birkmayer and Neumayer scale. | Improvement in clinical disability in all patients. |

**Published trials of NAD supplements in Parkinson's Disease.** Published studies of NAD supplements in Parkinson's Disease (n=6) have measured the effect of NR or NADH supplementation on clinical disability, cerebrospinal fluid metabolomics, as well as brain, urine, and blood NAD+ concentrations. The two recent studies of NR (post-2000) both found significant clinical improvement with NR supplementation.

**Table S3.**

| *NAD Supplements* | | | | |
|---|---|---|---|---|
| **Psychotic Disorders** | | | | |
| **Author** | **Intervention (type, dose, length)** | **Population (condition, n-enrolled subjects)** | **Primary Outcome(s)** | **Results (brief description)** |
| **Meltzer, Shader, & Grinspoon, 1969** *(118)* | 2g oral ***NAD*** for 21 days | **Schizoaffective disorder** (unknown sample size) | Clinical perceptions | NAD did not lead to gross clinical improvement; Increases in aggression, hostility, irritability observed in participants not taking concomitant phenothiazines. |
| **Kline, Barclay, & Esser, 1967** *(119)* | 1-2 g ***NAD***, 3-5 days | **Schizoaffective disorder** (n=17) | Clinical perceptions | 13 of 17 of cases showed "improvement." |
| **Ashby, Colins, & Bassett, 1960** *(120)* | nicotinic acid ***(NA)*** | **Schizophrenia** (n=39) | General clinical condition | No evidence of improvement in patients. |

**Published trials of NAD supplements in Psychotic Disorders.** Three studies of NAD supplementation in psychotic disorders have been published. However, there have been no published trials since 1969, and none of the existing trials used modern NAD supplements (e.g., NR, NMN) or reported results of statistical analyses.

**Table S4.**

| *NAD Supplements* | | | | |
|---|---|---|---|---|
| **Aging** | | | | |
| **Author** | **Intervention (type, dose, length)** | **Population (condition, n-enrolled subjects)** | **Primary Outcome(s)** | **Results (brief description)** |
| **Kuerec et al., 2024** *(51)* | ***NMN***, 300 mg, 600 mg, or 900 mg, 60 days | ***Aging***; treatment (n=20 per dosage group) vs. placebo (n=20) | Blood NAD+, blood biological age, HOMA-IR, 6-minute walking test, and SF-36 | Significant dose-dependent increase in blood NAD+ levels with high variability within groups (p<0.0001); The increase of NAD+ levels was associated with increases in walking distance and Short Form score. |
| **Akasaka et al., 2023** *(61)* | ***NMN***, 250 mg/day, 24 weeks | ***Aging and diabetes*** (n=14) | Safety, grip strength, and walking speed | Safe with no measured effects on grip strength or walking speed. |
| **Pencina et al., 2023** *(62)* | ***NMN,*** 500 mg/2x day, 28 days | ***Aging, obesity, and overweight***; treatment (n=20) vs placebo (n=8) | Safety, NAD+ concentration in blood and muscle | Blood total NAD increased ~200% at day 28 (p<0.05); Total NAD levels and aerobic capacity of muscle did not differ significantly assessed by 31P-MRS. |
| **Pencina et al., 2023** *(63)* | ***NMN,*** 1000 mg/day vs. 2000 mg/day vs. placebo, 14 days | ***Aging, obesity, and overweight***; treatment with 1000 mg (n=12) or 2000 mg (n=12) vs placebo (n=8) | Safety and blood NAD+ concentration | NMN supplementation was safe and increased blood total NAD concentrations by 1.7- and 3.7-fold for 1000 mg and 2000 mg groups, respectively, compared to placebo (p<0.001). |
| **Jensen et al., 2022** *(121)* | ***NR,*** 1000 mg NR and 200 mg PT, 22 days | ***Aging***; treatment (n=16) vs placebo (n=15) | Blood NAD+ and muscle injury recovery | Significant difference in blood NAD+ levels between treatment and placebo groups (p<0.05); Muscle NAD+ did not increase. |
| **Kim et al., 2022** *(60)* | ***NMN,*** 250 mg/day, 12 weeks | ***Aging*** (n=108) | Sleep quality, fatigue, and physical performance | NMN supplementation in the afternoon improved lower limb function (p=0.04) and fatigue (p<0.01). |
| **Yoshino et al., 2021** *(59)* | ***NMN,*** 250 mg/day, 8 weeks | ***Aging, post menopause, and prediabetes or obesity***; treatment (n=13) vs placebo (n=12) | Insulin stimulated glucose disposal and skeletal muscle insulin signaling | Increase in PBMC NAD+ compared to baseline and placebo (p<0.01). Increased insulin signaling measures (p<0.01) and upregulated genes related to muscle remodeling (p<0.05). |
| **Elhassan et al., 2019** *(122)* | ***NR,*** 1000 mg, 21 days | ***Aging*** (n=12) | Blood and muscle NAD+ and muscle metabolic function | Blood NAD+ increased more than 100% and blood NMN increased by 40% (p<0.001). Muscle mitochondrial function did not change, but metabolic gene transcription was altered. |

**Published trials of NAD supplements in Aging.** Eight trials of NAD supplementation in aging have been published, and the most common outcome measure is blood NAD+ concentration (n=5). NAD supplementation significantly increased blood NAD+ levels but did not influence muscle NAD+ levels.

## Table S5.

| *NAD Supplements - NR* | | | | |
|---|---|---|---|---|
| **Healthy controls (HC)** | | | | |
| **Author** | **Intervention (type, dose, length)** | **Population (condition, n-enrolled subjects)** | **Primary Outcome(s)** | **Results (brief description)** |
| **Nanga et al., 2024** *(100)* | ***NR,*** 900 mg, single dose | ***HC*** (n=10) | Cerebral NAD+ concentration measured by 7T MRS | Significantly increased cerebral NAD+ compared to baseline (p<0.001). |
| **Lapatto et al., 2023** *(146)* | ***NR***, 1000 mg, 150 days | ***HC*** (sixteen pairs of twins; n=32) | Whole blood NAD metabolite concentrations; change in muscle and white adipose tissue mitochondrial biogenesis | Increased whole blood concentrations of NAD+, NAAD, and NMN (p<0.001); Improved muscle mitochondrial biogenesis (p<0.05). |
| **Vreones et al., 2023** *(56)* | ***NR,*** 500 mg vs placebo, 2x/day, 6 weeks | ***HC***; treatment (n=12) vs placebo (n=10) | NAD+ concentration in plasma extracellular vesicles enriched for neuronal origin (NEVs) | Increased NAD+ levels in NEVs compared to placebo (p<0.001). AB42 in NEVs decreased in treatment responder subgroup (p=0.015), but not in overall population. |
| **Wu et al., 2022** *(147)* | ***NR***, 1000 mg, 7 days | ***HC*;** treatment (n=12) vs placebo (n=9) | Blood NAD+ concentrations, identification of NR-modulated immunometabolism genes | Altered regulation of autophagy and type I interferon signaling (p<0.05); 140% increase in whole blood NAD+ levels compared to placebo (p<0.0001). |
| **Conze, Brenner, & Kruger, 2019** *(48)* | ***NR,*** 100, 300, or 1000 mg, 8 weeks | ***HC*;** treatment with 100 mg (n=33), 300 mg (n=34), 1000 mg (n=32) vs placebo (n=34) | Blood NAD+ concentration | 100 mg - 22% increase (not significant), 300 mg – 51% increase (p<0.05), and 1000 mg – 142% (p<0.05) in blood NAD+ after 2 weeks compared to baseline and placebo. |
| **Martens et al., 2018** *(52)* | ***NR,*** 500 mg, 2x/day, 6 weeks | ***HC***; treatment (n=12) vs placebo (n=12) | Blood NAD+ concentration | Increased blood NAD+ by 60% compared to placebo (p<0.05). |
| **Airhart et al., 2017** *(50)* | ***NR,*** 250 mg days 1-2, 500 mg days 3-4, 1000 mg days 5-6, 2000 mg days 7-8 | ***HC*** (n=8) | Blood NAD+ concentration | Increased whole blood NAD+ concentration by 100% compared to baseline (p=0.001). |
| **Dellinger et al., 2017** *(49)* | ***NRPT (NR and pterostilbene),*** 250 mg and 50 mg or 500 mg and 100 mg, daily, 8 weeks | ***HC***; treatment with 250 mg (n=40), 500 mg (n=35) vs placebo (n=40) | Blood NAD+ concentration | Dose dependent blood NAD+ increase after 60 days compared to baseline; 250 mg – 40% (p<0.05) and 500 mg – 55% (p<0.05). |

**Published trials of NR supplements in Healthy Controls.** Eight trials of NAD supplementation in healthy adults have been published, and the most common outcome measure is blood NAD+ concentration (n=6). NR supplementation significantly increased blood NAD+ concentration, and two trials found a dose-dependent increase in blood NAD+ levels.

**Table S6.**

*NAD Supplements - NMN*

**Healthy controls (HC)**

| Author | Intervention (type, dose, length) | Population (condition, n-enrolled subjects) | Primary Outcome(s) | Results (brief description) |
|---|---|---|---|---|
| **Yamaguchi et al, 2024** *(148)* | ***NMN,*** *125 mg daily, 8 weeks* | ***HC*** (n=9) | Blood NAD+ concentration | PBMC NAD+ increased 63% compared to baseline after 8 weeks (p=0.0046). |
| **Morifuji et al., 2024** *(123)* | ***NMN,*** 250 mg, 84 days | ***HC*** (n=60) | Blood NAD+ concentration and sleep quality (PSQI) | Blood NAD+ increased 100% at week 4 (p<0.05) and 67% at week 12 (p<0.05) compared to placebo; Sleep quality improved (p=0.01). |
| **Yamane et al, 2023** *(124)* | ***NMN,*** 250 mg, 84 days | ***HC*** (n=11) | Blood NAD+ concentration | Blood NAD+ increased significantly at 1, 2, and 3 months (p<0.05). |
| **Katayoshi et al., 2023** ***(149)*** | ***NMN***, 250 mg/day, 12 weeks | ***Healthy, middle-aged adults*** (n=36) | Serum NAD+ concentrations. | 57.6% increase in serum NAD+ concentrations compared to baseline (p=0.037). |
| **Yi et al., 2023** *(150)* | ***NMN***, 300, 600, or 900 mg/day, 60 days | ***HC***; treatment (n=20 per dosage group vs placebo (n=20) | Total blood NAD concentration | Dose-dependent increase in blood total NAD concentrations for all NMN-treated groups (p<0.001). |
| **Igarashi et al., 2022** *(125)* | ***NMN,*** 250 mg/day, 12 weeks | ***HC***; treatment (n=10) vs placebo (n=10) | Blood NAD+ and muscle function | Whole blood NAD+ was significantly increased compared to placebo (p<0.001). Note: concentrations of NAD+ at baseline (measured in uM) are inconsistent with previous reports. |
| **Okabe et al., 2022** *(126)* | ***NMN,*** 250 mg/day, 12 weeks | ***HC*** (n=15) | Blood NAD+ and related metabolites | Whole blood NAD+ increased significantly at 4, 8 and 12 weeks compared to baseline (p<0.001). |
| **Huang, 2022** *(127)* | ***NMN***, 300 mg/day, 60 days | ***HC***; treatment (n=31) vs placebo (n=31) | Blood NAD+/NADH, HOMA-IR | Serum NAD+/NADH increased 38% compared to baseline (p=0.10). No significant difference compared to placebo (p=0.40). No significant change in HOMA-IR in both groups. |
| **Kimura et al., 2022** *(128)* | ***NMN***, 300 mg, intravenous, measured for 5 hours | ***HC*** (n=10) | Blood NAD+/NADH, EKG, pulse, blood pressure | Blood NAD+ increased 20%; NAD+/NADH decreased 10% (not significant after 5 hours, p>0.05). |
| **Irie et al., 2020** *(130)* | ***NMN,*** 100, 300, or 500, single dose | ***HC*** (n=10) | Plasma NMN metabolites, safety | Safe and well tolerated. Increase in plasma concentrations of N-methyl-2-pyridone-<br>5-carboxamide (2Py) and 4Py (p<0.01). |

**Published trials of NMN supplements in Healthy Controls.** Ten trials of NMN supplementation in healthy adults have been published, and the most common outcome measure is blood NAD concentration (n=9). NMN supplementation significantly increased blood NAD+ concentration, and one trial found a dose-dependent increase in blood NAD+ levels.

## Table S7.

*Ketogenic Therapy*

**AD and MCI**

| Author | Intervention (type, dose, length) | Population (condition, n-enrolled subjects) | Primary Outcome(s) | Results (brief description) |
|---|---|---|---|---|
| **Buchholz et al., 2024** *(131)* | ***Modified Atkins Diet***; 12 weeks. | ***AD***; treatment (n=20) vs control diet (n=18) | Cognition, plasma metabolomic analysis - baseline and 12 weeks | 2-3x increase in memory in treatment group compared to control group (medium effect size, no p-value reported); 13 metabolites and 10 lipids showed significant change from baseline (p<0.05). |
| **Hanson et al., 2023** *(132)* | Saline and ***triglyceride infusions*** (44 cc triglyceride emulsion/hour; 5 hours) | ***Cognitive impairment*** (n=9) vs cognitively normal (n=12) | 215 aqueous metabolites from 35 metabolic pathways in CSF | Significant increase in CSF 3-hydroxybutyrate (p=0.003); Increases were larger in cognitive impairment group compared to cognitively normal (p=0.02). |
| **Kumar et al., 2022** *(81)* | Modified ***Mediterranean Ketogenic Diet***; 6 weeks | Cognitively normal (n=11) vs ***MCI*** (n=9) | Amyloid beta 1-42, p181 tau, and neurofilament light in plasma small extracellular vesicles | Reduced amyloid beta 1-42 (p=0.011), p181 tau (p=0.033), and neurofilament light (p=0.020) in MCI participants. |
| **Roy at el., 2022** *(77)* | ***MCT drink*** (30 g/day); 6 months | ***MCI***; treatment (n=17) vs placebo (n=15) | Dorsal attention network (DAN) functional connectivity | Improved dorsal attention network functional connectivity (p=0.024); Functional connectivity was associated with improved attention (p=0.021). |
| **Phillips et al., 2021** *(133)* | ***Ketogenic diet vs. usual diet with healthy eating guideline***s; 12 weeks. | Probable ***AD***; treatment (n=18) vs usual diet (n=18) | Cognition, daily living, quality of life, measured at baseline, 6 weeks, and 12 weeks | Daily living (p=0.0067) and quality of life improved (p=0.023). No significant change in cognition (p=0.24). Significant difference in blood beta-hydroxybutyrate between groups (p<0.001). |
| **Myette-Cote et al., 2021** *(82)* | ***MCT drink*** (30 g/day)or calorie-match placebo (high oleic acid); 6 months | ***MCI***; treatment (n=19) vs placebo (n=20) | Plasma cardiometabolic and inflammatory markers | Significant increase in plasma ketones in the kMCT group (p<0.001); Significant increase in circulating IL-8 (p=0.002). |
| **Roy et al., 2021** *(134)* | ketogenic medium chain triglycerides ***(kMCT) drink***, 30 g/day; 6 months | ***MCI***; placebo (n=15) vs treatment (n=17) | Cognition, PET - ketone and glucose update, DTI - structural properties of white matter | Increased cortical (p=0.006) and white matter (p<0.001) ketone uptake in kMCT group; Improvement in processing speed (p=0.017) and increased dorsal attention network functional connectivity (p=0.035) were associated with WM ketone uptake. |

| | | | | |
|---|---|---|---|---|
| **Fortier et al., 2021** *(78)* | ***Ketogenic drink*** containing medium chain triglyceride - 15g 2x per day; 6 months | ***MCI***; treatment (n =39) vs placebo (n=39) | Cognition, plasma ketone levels | Recall (p=0.047), verbal fluency (p=0.024), language (p=0.033) and executive function test scores (p=0.017) improved in treatment group compared to placebo; Cognitive outcomes positively correlated with plasma ketones (p<0.05); Total plasma ketones increased significantly compared to placebo (p<0.0001). |
| **Ota et al., 2019** *(135)* | ***MCT-based ketogenic diet***, 50 g ketogenic formula (20 g MCTs); 12 weeks | ***Mild to moderate AD*** (n=16) | Cognition, plasma ketone levels | No significant change in plasma ketones from baseline; Logical memory (p<0.05) and processing speed (p<0.05) increased significantly from baseline. |
| **Fortier et al., 2019** *(76)* | ***MCT drink*** (30 g/day); 6 months | ***MCI***; treatment (n=19) vs placebo (n=20); | PET - brain ketone and glucose metabolism, cognition; baseline and 6 months | Brain ketone metabolism increased 230% for the MCT group (p<0.001); No change in glucose metabolism (p>0.1); Improved cognition in MCT group (p<0.05); Direct relationship between improved cognition and increased plasma ketones (p>0.05). |
| **Torosyan et al., 2018** *(136)* | ***Caprylidene (MCT)***, 40 g/day; 45 days | ***Mild to moderate AD*** with and without E4 allele; treatment (n=14) vs placebo (n=2) | 150-water PET - Regional cerebral blood flow | Subjects without E4 allele had significant elevation of regional cerebral blood flow in the left superior lateral temporal cortex (p=0.04). No change in subjects with E4 allele. |
| **Croteau et al., 2018.** *(79)* | ***Two MCT*** **supplements** administered separately (both 30 g/day); one month each | ***AD*** (n=15); 7/15 took the second ketogenic supplement after a 4-week washout period | PET - brain ketone and glucose metabolism | Both MCT supplements increased total brain ketone (p=0.046) metabolism without affecting brain glucose utilization (p=0.657). |
| **Taylor et al., 2018** *(151)* | ***Ketogenic diet*** and ***MCT supplement*** (MCT oil); 12 weeks | ***AD*** (n=10) | Serum ketone concentrations, safety and feasibility | Increase in serum BHB after 4, 8, and 12 weeks (p<0.001) compared to baseline. |
| **Krikorian et al., 2012** *(152)* | ***Ketogenic diet*** vs high carbohydrate diet, 6 weeks | ***MCI***; ketogenic (n=12) vs high carb (n=11) diet | Executive function, memory, mood, urine ketone and glucose | Improved verbal memory (p=0.01); ketone concentrations positively correlated with memory (p=0.04). |
| **Henderson et al., 2009** *(80)* | AC-1202 (oral ***ketogenic*** compound); 90-days | ***Mild-to-moderate AD***; treatment (n=77) vs placebo (n=63) | Cognition and clinical change over time, plasma ketone levels | Increase in serum BHB levels compared to placebo (p<0.0001). In non APOE4 subjects, cognition improved from baseline; significant interaction between serum BHB levels and cognition in non APOE4 subjects. |

**Published trials of ketogenic therapy in AD and MCI**. Fifteen trials of ketogenic therapy in AD and MCI have been conducted. Seven trials found improved cognitive performance after ketogenic therapy. Additionally, multiple trials found increases in brain ketone levels without altering brain glucose utilization using PET.

## Table S8.

| *Ketogenic Therapy* | | | | |
|---|---|---|---|---|
| **Parkinson's Disease** | | | | |
| **Author** | **Intervention (type, dose, length)** | **Population (condition, n-enrolled subjects)** | **Primary Outcome(s)** | **Author** |
| **Choi et al., 2024** *(83)* | ***Ketogenic diet*** with MCT oil supplement (MCT-KD); 3 weeks | ***Parkinson's Disease*** ; treatment (n=7) vs standard diet (n=9) | Acceptability and Feasibility, Timed Up and Go (TUG) mobility test at day 7 | Increase in plasma ketones at 3 weeks (no reported statistic); no change in TUG at day 7; Nonmotor symptom severity reduced in MCT-KD group (p=0.04). |
| **Tidman, White, & White, 2022** *(84)* | ***Ketogenic diet***, 12 weeks | ***Parkinson's Disease*** (n=16) | UPDRS, Parkinson's Anxiety Score (PAS), Center for Epidemiologic Studies Depression Scale Revised-20 (CESD-R-20) | Improvements in PAS scores (p=0.00086), Part I of the UPDRS (p=0.00079) compared to baseline. No significant differences in CESD-R-20 compared to baseline (p=0.46). |
| **Koyuncu et al., 2021** *(85)* | ***Ketogenic diet*** vs regular diet; 3 months | ***Parkinson's Disease with voice disorder***; treatment (n=34) vs regular diet (n=34) | Voice Handicap Index (VHI) | All VHI parameters improved in the KD group (p<0.001), but no VHI parameters significantly improved in the regular diet group . |
| **Phillips et al., 2018** *(86)* | ***Ketogenic diet*** or low fat, high carb diet; 8 weeks | ***Parkinson's Disease***; KD (n=18) vs low-fat diet (n=20) | UPDRS Parts 1-4 scores, blood glucose and ketone concentrations | Both groups had decreased UPDRS scores, but the KD group decreased 41% (p<0.001) while the low fat group had a 11% decrease (p=0.03) in part 1; blood glucose decreased (p=0.001) and blood ketones increased (p<0.001) in KD compared to low-fat group. |
| **Vanitallie et al., 2005** *(153)* | ***Ketogenic diet***, 28 days | ***Parkinson Disease***, n=5 | UPDRS scores | UPDRS scores improved in all five participants during hyperketonemia (no reported p value or statistics). |

**Published trials of ketogenic therapy in Parkinson's Disease**. Five trials of ketogenic therapy in Parkinson's Disease have been conducted. Four of five trials found improvements in clinical symptoms (measured by the UPDRS) during the ketogenic diet.

## Table S9.

*Ketogenic Therapy*

**Psychotic and Bipolar Disorders**

| Author | Intervention (type, dose, length) | Population (condition, n-enrolled subjects) | Primary outcome(s) | Results (brief summary) |
|---|---|---|---|---|
| **Campbell et al., 2025** *(88)* | ***Modified ketogenic diet***, 6-8 weeks | ***Bipolar disorder*** (n=20) | Metabolic health, clinical scales, glucose, ketone, and TCA-related metabolite levels, MRS | Decreased body weight (p<0.001) and systolic blood pressure(p<0.041). Increase in serum BHB levels (p<0.001), and decreased lactate levels (p=0.017). Significant correlation between ketone levels and improved self-rated mood, energy, impulsivity and anxiety (p<0.001). Decrease in MRS glutamine + glutamate concentrations in ACC (p=0.025) and PCC (p<0.001). |
| **Sethi et al., 2024** ***(75)*** | ***Ketogenic diet***, 4 months | ***Bipolar disorder I and II, schizophrenia, schizoaffective disorder*** (n=21) | HOMA-IR, triglyceride levels, Brief Psychiatric Rating Scale (BPRS), CGI | 27% reduction in HOMA-IR (p<0.05) 25% decrease in triglyceride levels (p<0.05), and 79% decrease in CGI scores (p<0.001). |
| **Danan et al., 2022** *(89)* | ***Ketogenic diet***, range from 14 days to 248 days | ***Bipolar, major depressive, schizoaffective disorders (inpatient)*** *(n=28)* | Clinical scales for depression (HAM-D, MADRS), PANSS, clinical global improvement (CGI), metabolic health | Significant improvement in depression, PANSS, and CGI scores (p<0.001); Improvements in weight (p<0.001), blood pressure (p<0.001), blood glucose (p=0.003), and triglycerides (p=0.003). |
| **Chmiel, 2022** *(138)* | ***Low-fat, high carbohydrate diet***, 1 year, **ketogenic diet**, 1 year, ***ketogenic diet with fasting 1x/week***, 1 year | ***Bipolar disorder with rapid cycling*** (n=1) | Blood ketone levels, clinical impressions | Increase in blood ketone concentrations; improvement in energy, sleep, cognition, anxiety, and mood stability. |
| **Saraga et al., 2020** *(139)* | ***Ketogenic diet***, unknown duration | ***Bipolar I disorder*** (n=1) | Urine ketone concentrations, clinical impressions | Achieved ketosis; improved anxiety and manic/depressive symptoms |
| **Palmer, Gilbert-Jaramillo, & Westman, 2019** *(140)* | ***Ketogenic diet***, several months, 5 years | ***Schizophrenia*** (n=2) | PANSS, patient report, weight | Reduction in PANSS scores. Reductions in body weight. |
| **Gilbert-Jaramillo et al., 2018** *(141)* | ***Ketogenic diet***, 6 weeks | ***Schizophrenia*** (n=2) | PANSS, blood tests, weight | Reductions in PANSS scores, body weight, and liver enzymes in 1 patient. |
| **Palmer, 2017** *(142)* | ***Ketogenic diet***, 4 months and 12 months | ***Schizoaffective disorder*** (n=2) | PANSS, patient report, weight | Reduction in PANSS scores. Improved energy, mood, concentration. Reduced weight. |
| **Phelps et al., 2013** *(143)* | ***Ketogenic diet***, 1 year or 2 years | ***Bipolar II disorder*** (n=2) | Urinary ketone concentrations, clinical impressions | Increased urine ketone concentrations; mood stability; decreased anxiety. |

| | | | | |
|---|---|---|---|---|
| **Kraft & Westman, 2009** *(144)* | ***Ketogenic diet***, 12 months | ***Schizophrenia*** (n=1) | Patient report | Auditory and visual hallucinations remitted. |
| **Yaroslavsky et al., 2002** *(145)* | ***Ketogenic diet***, 2 weeks, ***MCT oil***, 2 weeks | ***Bipolar I disorder*** (n=1) | Urine ketone concentrations, clinical impressions | Suspected non-compliance with diet; no increase in urine ketone concentrations; no clinical improvement. |
| **Pacheco, Easterling, & Prior, 1965** *(137)* | ***Ketogenic diet***, 2 weeks | ***Schizophrenia*** *(n=10)* | Beckomberga Rating Scale for the S-Factor, clinical perceptions | A decrease in Beckomberga scores was observed for all patients evidencing clinical improvement (no reported p-value). |

**Published trials of ketogenic therapy in Psychotic and Bipolar Disorders**. Twelve trials of ketogenic therapy in psychotic or bipolar disorders have been conducted. Only four trials had a sample size greater than two, and three of these four trials found a significant improvement in clinical symptoms.

**Table S10.**

| NR (Healthy controls) | | | | | | | | |
|---|---|---|---|---|---|---|---|---|
| **Author** | **Year published** | **Dose (mg) per day** | **Days** | **Age range in years (average)** | **Sample size receiving treatment** | **% Change in whole blood NAD+** | **p-value** | **Notes** |
| **Lapatto et al.** *(146)* | 2023 | 1000 | 150 | 30-45 (40) | 32 | 130% | <0.001 | Crossover, placebo controlled. |
| **Wu et al.** *(147)* | 2022 | 1000 | 7 | 18-39 (24) | 41 | 140% | <0.0001 | |
| **Elhassan et al.** *(122)* | 2019 | 1000 | 21 | 70-80 (75) | 12 | 170% | <0.001 | |
| **Conze et al.*** *(48)* | 2019 | 100 | 56 | 40-60 (52) | 33 | 10% | >0.05 | |
| **Conze et al.*** *(48)* | 2019 | 300 | 56 | 40-60 (50) | 34 | 48% | <0.05 | |
| **Conze et al.*** *(48)* | 2019 | 1000 | 56 | 40-60 (51) | 32 | 139% | <0.05 | |
| **Martens et al.** *(52)* | 2018 | 1000 | 42 | 55-79 (65) | 12 | 60% | <0.05 | |
| **Airhart et al.** *(50)* | 2017 | 687.5 | 8 | 21-50 (33) | 8 | 100% | 0.001 | Average dose. Dose was increased every 2 days |
| **Dellinger et al.*** *(49)* | 2017 | 250 | 60 | 60-80 (68) | 40 | 40% | <0.05 | With 50 mg of pterostilbene |
| **Dellinger et al.*** *(49)* | 2017 | 500 | 60 | 60-80 (66) | 35 | 55% | <0.05 | With 100 mg of pterostilbene |

**Published trials of NR supplements and their effects on blood NAD+ levels in healthy controls.**

*Multiple time points from the same sample are included in this table.

**Table S11.**

| NR (patients) | | | | | | | | |
|---|---|---|---|---|---|---|---|---|
| **Author** | **Year published** | **Dose (mg) per day** | **Days** | **Age range in years (average)** | **Sample size receiving treatment** | **% Change in whole blood NAD+** | **p-value** | **Notes** |
| **Orr et al.** *(53)* | 2024 | 1000 | 70 | 67-86 (77) | 10 | 160% | <0.001 | MCI |
| **Berven et al.** *(57)* | 2023 | 3000 | 28 | 35-100 (61) | 10 | 270% | <0.001 | PD |
| **Jensen et al.** *(121)* | 2022 | 1000 | 22 | 55-80 (69) | 16 | 350% | <0.05 | Induced muscle injury. Blood NAD+ levels estimated from graph. |

**Published trials of NR supplements and their effects on blood NAD+ levels in patients.**

**Table S12.**

| NMN (Healthy controls) | | | | | | | | |
|---|---|---|---|---|---|---|---|---|
| **Author** | **Year published** | **Dose (mg) per day** | **Days** | **Age range in years (average)** | **Sample size receiving treatment** | **% Change in whole blood NAD+** | **p-value** | **Notes** |
| **Yamaguchi et al.*** *(148)* | 2024 | 250 | 7 | 40-60 (48) | 9 | 17% | Not reported | PBMC |
| **Yamaguchi et al.*** *(148)* | 2024 | 250 | 14 | 40-60 (48) | 9 | 35% | Not reported | |
| **Yamaguchi et al.*** *(148)* | 2024 | 250 | 28 | 40-60 (48) | 9 | 47% | Not reported | |
| **Yamaguchi et al.*** *(148)* | 2024 | 250 | 56 | 40-60 (48) | 9 | 63% | 0.0046 | |
| **Morifuji et al.*** *(123)* | 2024 | 250 | 28 | 65-70 (69) | 9 | 100% | <0.05 | Whole blood NAD+ |
| **Morifuji et al.*** *(123)* | 2024 | 250 | 84 | 65-70 (69) | 9 | 67% | <0.05 | Whole blood NAD+ |
| **Kuerec et al.*,**** *(51)* | 2024 | 300 | 30 | 40-65 (50) | 20 | N/A | <0.0001 | 240% increase in total blood NAD. |
| **Kuerec et al.*,**** *(51)* | 2024 | 300 | 60 | 40-65 (50) | 20 | N/A | <0.0001 | 277% increase in total blood NAD. |
| **Kuerec et al.*,**** *(51)* | 2024 | 600 | 30 | 40-65 (50) | 20 | N/A | <0.0001 | 415% increase in total blood NAD. |
| **Kuerec et al.*,**** *(51)* | 2024 | 600 | 60 | 40-65 (50) | 20 | N/A | <0.0001 | 499% increase in total blood NAD. |
| **Kuerec et al.*,**** *(51)* | 2024 | 900 | 30 | 40-65 (50) | 20 | N/A | <0.0001 | 436% increase in total blood NAD. |
| **Kuerec et al.*,**** *(51)* | 2024 | 900 | 60 | 40-65 (50) | 20 | N/A | <0.0001 | 508% increase in total blood NAD. |
| **Yi et al.**** *(150)* | 2023 | 300 | 60 | 40-65 (51.2) | 20 | N/A | <0.001 | 176% increase in total blood NAD. |
| **Yi et al.**** *(150)* | 2023 | 600 | 60 | 40-65 (49.5) | 20 | N/A | <0.001 | 470% increase in total serum NAD |
| **Yi et al.**** *(150)* | 2023 | 900 | 60 | 40-65 (49.9) | 20 | N/A | <0.001 | 362% increase in total blood NAD |
| **Yamane et al.**** *(124)* | 2023 | 250 | 84 | 20-65 (46.1) | 11 | N/A | <0.05 | 317% increase in plasma NAD+ levels; NAD+ levels estimated from graph. |
| **Pencina et al.** *(62)* | 2023 | 2000 | 28 | >45 (61.9) | 20 | 175% | <0.05 | Obesity and overweight. Blood NAD+ increase estimated from graph. |
| **Pencina et al. **** *(63)* | 2023 | 1000 | 14 | 55-80 (63.9) | 12 | N/A | <0.001 | 100% increase in blood total NAD levels; NAD levels estimated from graph. |
| **Pencina et al. **** *(63)* | 2023 | 2000 | 14 | 55-80 (63.9) | 12 | N/A | <0.001 | 150% increase in blood total NAD |

| | | | | | | | | |
|---|---|---|---|---|---|---|---|---|
| | | | | | | | | levels; NAD levels estimated from graph. |
| **Katayoshi et al.**** *(149)* | 2023 | 250 | 84 | 40-65 (48) | 36 | N/A | 0.037 | 57.6% increase in serum NAD+ concentrations compared to baseline. |
| **Okabe et al.** *(126)* | 2022 | 250 | 84 | 22-64 (43.9) | 15 | 100% | <0.001 | Whole blood NAD+ levels estimated from graph. |
| **Huang et al.**** *(127)* | 2022 | 300 | 60 | 40-65 (47.7) | 31 | N/A | 0.10 | 38% increase in blood NAD+/NADH ratio |
| **Kimura et al.**** *(128)* | 2022 | 300 | 0.21 | 20-70 (43.4) | 10 | 20% | >0.05 | IV infusion; blood NAD+ levels estimated from graph. |
| **Igarashi et al.**** *(125)* | 2022 | 250 | 84 | 65+ (69.8) | 10 | 508% | >0.001 | Reported differences in whole blood NAD+ in placebo vs. treatment group but not baseline vs. 12-week. Baseline whole blood NAD+ concentrations inconsistent with all previous reports. |

**Published trials of NMN supplements and their effects on blood NAD+ levels in healthy controls.**

*Multiple time points from the same sample are included in this table.

**Excluded from Figure 2 due to the type of NAD measurement used. These studies may have measured total NAD or NAD/NADH ratio rather than NAD+ concentrations. Additional studies were excluded due to the measurement of plasma NAD+ concentrations rather than whole blood.

**Table S13.**

| NMN (patients) | | | | | | | | |
|---|---|---|---|---|---|---|---|---|
| **Author** | **Year published** | **Dose (mg) per day** | **Days** | **Age range in years (average)** | **Sample size receiving treatment** | **% Change in whole blood NAD+** | **p-value** | **Notes** |
| **Yoshino et al.** *(59)* | 2021 | 250 | 70 | (62) | 13 | N/A | <0.01 | Postmenopausal women with prediabetes. 68% increase in PBMC NAD+ compared to baseline. PBMC NAD+ change estimated from graph |

**Published trials of NMN supplements and their effects on blood NAD+ levels in healthy controls.**

## REFERENCES


1. Alzheimer's Association Report, 2023 Alzheimer's disease facts and figures. *Alzheimers Dement.* **19**, 1598–1695 (2023).

2. L. M. Waite, New and emerging drug therapies for Alzheimer disease. *Aust. Prescr.* **47**, 75–79 (2024).

3. S. C. Cunnane, E. Trushina, C. Morland, A. Prigione, G. Casadesus, Z. B. Andrews, M. F. Beal, L. H. Bergersen, R. D. Brinton, S. de la Monte, A. Eckert, J. Harvey, R. Jeggo, J. H. Jhamandas, O. Kann, C. M. la Cour, W. F. Martin, G. Mithieux, P. I. Moreira, M. P. Murphy, K. A. Nave, T. Nuriel, S. H. R. Oliet, F. Saudou, M. P. Mattson, R. H. Swerdlow, M. J. Millan, Brain energy rescue: An emerging therapeutic concept for neurodegenerative disorders of ageing. *Nat. Rev. Drug Discov.* **19**, 609–633 (2020).

4. B. M. Kuehn, In Alzheimer research, glucose metabolism moves to center stage. *JAMA* **323**, 297–299 (2020).

5. S. Lautrup, D. A. Sinclair, M. P. Mattson, E. F. Fang, $NAD^{+}$ in brain aging and neurodegenerative disorders. *Cell Metab.* **30**, 630–655 (2019).

6. P. Stępnicki, M. Kondej, A. A. Kaczor, Current concepts and treatments of schizophrenia. *Molecules* **23**, 2087 (2018).

7. R. A. McCutcheon, R. S. E. Keefe, P. K. McGuire, Cognitive impairment in schizophrenia: Aetiology, pathophysiology, and treatment. *Mol. Psychiat.* **28**, 1902–1918 (2023).

8. D. Ben-Shachar, D. Laifenfeld, Mitochondria, synaptic plasticity, and schizophrenia. *Int. Rev. Neurobiol.* **59**, 273–296 (2004).

9. F. Du, A. J. Cooper, T. Thida, S. Sehovic, S. E. Lukas, B. M. Cohen, X. Zhang, D. Ongur, In vivo evidence for cerebral bioenergetic abnormalities in schizophrenia measured using 31P magnetization transfer spectroscopy. *JAMA Psychiat.* **71**, 19–27 (2014).

10. E. Ling, J. Nemesh, M. Goldman, N. Kamitaki, N. Reed, R. E. Handsaker, G. Genovese, J. S. Vogelgsang, S. Gerges, S. Kashin, S. Ghosh, J. M. Esposito, K. Morris, D. Meyer, A. Lutservitz, C. D. Mullally, A. Wysoker, L. Spina, A. Neumann, M. Hogan, K. Ichihara, S. Berretta, S. A. McCarroll, A concerted neuron-astrocyte program declines in ageing and schizophrenia. *Nature* **627**, 604–611 (2024).

11. L. Rajman, K. Chwalek, D. A. Sinclair, Therapeutic potential of NAD-boosting molecules: The in vivo evidence. *Cell Metab.* **27**, 529–547 (2018).

12. G. E. Hardingham, K. Q. Do, Linking early-life NMDAR hypofunction and oxidative stress in schizophrenia pathogenesis. *Nat. Rev. Neurosci.* **17**, 125–134 (2016).

13. F. M. Fitzpatrick, N. Kory, Guardians of the cell: Mitochondria as a rheostat for cellular $NAD^+$ levels. *Nat. Metab.* **6**, 2215–2217 (2024).

14. M. V. Damgaard, J. T. Treebak, What is really known about the effects of nicotinamide riboside supplementation in humans. *Sci. Adv.* **9**, eadi4862 (2023).

15. L. Xin, Ö. Ipek, M. Beaumont, M. Shevlyakova, N. Christinat, M. Masoodi, N. Greenberg, R. Gruetter, B. Cuenoud, Nutritional ketosis increases $NAD^+$/NADH ratio in healthy human brain: An in vivo study by 31P-MRS. *Front. Nutr.* **5**, 62 (2018).

16. E. Ozan, V.-A. Chouinard, C. M. Palmer, The ketogenic diet as a treatment for mood disorders. *Curr. Treat. Options Psych.* **11**, 163–176 (2024).

17. R. Dringen, Metabolism and functions of glutathione in brain. *Prog. Neurobiol.* **62**, 649–671 (2000).

18. V.-A. Chouinard, W. Feizi, X. Chen, B. Ren, K. E. Lewandowski, J. Anderson, S. Prete, E. Tusuzian, K. Cuklanz, S. Zhou, P. Bolton, A. Stein, B. M. Cohen, F. du, D. Öngür, Intranasal insulin increases brain glutathione (GSH) and enhances antioxidant capacity in healthy participants, but not in those with early psychotic disorders. *Biol. Psychiatry Cogn. Neurosci. Neuroimaging* **10**, 286–294 (2025).

19. N. Braidy, Y. Liu, NAD$^+$ therapy in age-related degenerative disorders: A benefit/risk analysis. *Exp. Gerontol.* **132**, 110831 (2020).

20. V. Kumar, S. H. Kim, K. Bishayee, Dysfunctional glucose metabolism in Alzheimer's disease onset and potential pharmacological interventions. *Int. J. Mol. Sci.* **23**, 9540 (2022).

21. A. Drzezga, N. Lautenschlager, H. Siebner, M. Riemenschneider, F. Willoch, S. Minoshima, M. Schwaiger, A. Kurz, Cerebral metabolic changes accompanying conversion of mild cognitive impairment into Alzheimer's disease: A PET follow-up study. *Eur. J. Nucl. Med. Mol. Imaging* **30**, 1104–1113 (2003).

22. M. M. I. Abdalla, Insulin resistance as the molecular link between diabetes and Alzheimer's disease. *World J. Diabetes* **15**, 1430–1447 (2024).

23. V.-A. Chouinard, D. C. Henderson, C. Dalla Man, L. Valeri, B. E. Gray, K. P. Ryan, A. M. Cypess, C. Cobelli, B. M. Cohen, D. Öngür, Impaired insulin signaling in unaffected siblings and patients with first-episode psychosis. *Mol. Psychiatry* **24**, 1513–1522 (2019).

24. A. Stein, C. Zhu, F. Du, D. Öngür, Magnetic resonance spectroscopy studies of brain energy metabolism in schizophrenia: Progression from prodrome to chronic psychosis. *Curr. Psychiatry Rep.* **25**, 659–669 (2023).

25. B. I. Aldana, Microglia-specific metabolic changes in neurodegeneration. *J. Mol. Biol.* **431**, 1830–1842 (2019).

26. N. R. Cleland, S. I. Al-Juboori, E. Dobrinskikh, K. D. Bruce, Altered substrate metabolism in neurodegenerative disease: New insights from metabolic imaging. *J. Neuroinflammation* **18**, 1–18 (2021).

27. L. Rowland, S. Pradhan, S. Korenic, S. Wijtenburg, L. Hong, R. Edden, P. Barker, Elevated brain lactate in schizophrenia: A 7 T magnetic resonance spectroscopy study. *Transl. Psychiatry* **6**, e967 (2016).

28. X. Song, X. Chen, C. Yuksel, J. Yuan, D. A. Pizzagalli, B. Forester, D. Ongur, F. Du, Bioenergetics and abnormal functional connectivity in psychotic disorders. *Mol. Psychiatry* **26**, 2483–2492 (2021).

29. S. Y. Kim, B. M. Cohen, X. Chen, S. E. Lukas, A. K. Shinn, A. C. Yuksel, T. Li, F. Du, D. Ongur, Redox dysregulation in schizophrenia revealed by in vivo $NAD^+$/NADH measurement. *Schizophr. Bull.* **43**, 197–204 (2017).

30. J. J. Harris, R. Jolivet, D. Attwell, Synaptic energy use and supply. *Neuron* **75**, 762–777 (2012).

31. L. A. Voloboueva, R. G. Giffard, Inflammation, mitochondria, and the inhibition of adult neurogenesis. *J. Neurosci. Res.* **89**, 1989–1996 (2011).

32. J. J. Iliff, M. Wang, Y. Liao, B. A. Plogg, W. Peng, G. A. Gundersen, H. Benveniste, G. E. Vates, R. Deane, S. A. Goldman, E. A. Nagelhus, M. Nedergaard, A paravascular pathway facilitates CSF flow through the brain parenchyma and the clearance of interstitial solutes, including amyloid β. *Sci. Transl. Med.* **4**, 147ra111 (2012).

33. Y.-H. Chen, S. Lin, S.-Y. Jin, T.-M. Gao, Extracellular ATP is a homeostatic messenger that mediates cell–cell communication in physiological processes and psychiatric diseases. *Biol. Psychiatry* **97**, 41–53 (2025).

34. B. S. Khakh, G. Burnstock, The double life of ATP. *Sci. Am.* **301**, 84–90 (2009).

35. S. Imai, L. Guarente, $NAD^+$ and sirtuins in aging and disease. *Trends Cell Biol.* **24**, 464–471 (2014).

36. X.-H. Zhu, M. Lu, B.-Y. Lee, K. Ugurbil, W. Chen, In vivo NAD assay reveals the intracellular NAD contents and redox state in healthy human brain and their age dependences. *Proc. Natl. Acad. Sci. U.S.A.* **112**, 2876–2881 (2015).

37. M. R. McReynolds, K. Chellappa, J. A. Baur, Age-related $NAD^+$ decline. *Exp. Gerontol.* **134**, 110888 (2020).

38. A. J. Covarrubias, R. Perrone, A. Grozio, E. Verdin, $NAD^+$ metabolism and its roles in cellular processes during ageing. *Nat. Rev. Mol. Cell Biol.* **22**, 119–141 (2021).

39. K. J. Barnham, C. L. Masters, A. I. Bush, Neurodegenerative diseases and oxidative stress. *Nat. Rev. Drug Discov.* **3**, 205–214 (2004).

40. B. Poljsak, V. Kovac, S. Spalj, I. Milisav, The central role of the $NAD^+$ molecule in the development of aging and the prevention of chronic age-related diseases: Strategies for $NAD^+$ modulation. *Int. J. Mol. Sci.* **24**, 2959 (2023).

41. I. G. Onyango, Modulation of mitochondrial bioenergetics as a therapeutic strategy in Alzheimer's disease. *Neural Regen. Res.* **13**, 19–25 (2018).

42. D. Dwir, I. Khadimallah, L. Xin, M. Rahman, F. Du, D. Ongur, K. Q. Do, Redox and immune signaling in schizophrenia: New therapeutic potential. *Int. J. Neuropsychopharmacol.* **26**, 309–321 (2023).

43. T. Iqbal, T. Nakagawa, The therapeutic perspective of $NAD^+$ precursors in age-related diseases. *Biochem. Biophys. Res. Commun.* **702**, 149590 (2024).

44. X. A. Cambronne, W. L. Kraus, Location, location, location: Compartmentalization of $NAD^+$ synthesis and functions in mammalian cells. *Trends Biochem. Sci.* **45**, 858–873 (2020).

45. M. E. Migaud, M. Ziegler, J. A. Baur, Regulation of and challenges in targeting $NAD^+$ metabolism. *Nat. Rev. Mol. Cell Biol.* **25**, 822–840 (2024).

46. J. She, R. Sheng, Z.-H. Qin, Pharmacology and potential implications of nicotinamide adenine dinucleotide precursors. *Aging Dis.* **12**, 1879 (2021).

47. N. Xie, L. Zhang, W. Gao, C. Huang, P. E. Huber, X. Zhou, C. Li, G. Shen, B. Zou, $NAD^+$ metabolism: Pathophysiologic mechanisms and therapeutic potential. *Signal Transduct. Target. Ther.* **5**, 227 (2020).

48. D. Conze, C. Brenner, C. L. Kruger, Safety and metabolism of long-term administration of NIAGEN (nicotinamide riboside chloride) in a randomized, double-blind, placebo controlled clinical trial of healthy overweight adults. *Sci. Rep.* **9**, 9772 (2019).

49. R. W. Dellinger, S. R. Santos, M. Morris, M. Evans, D. Alminana, L. Guarente, E. Marcotulli, Repeat dose NRPT (nicotinamide riboside and pterostilbene) increases $NAD^+$ levels in humans safely and sustainably: A randomized, double-blind, placebo-controlled study. *NPJ Aging Mech. Dis.* **3**, 17 (2017).

50. S. E. Airhart, L. M. Shireman, L. J. Risler, G. D. Anderson, G. Nagana Gowda, D. Raftery, R. Tian, D. D. Shen, K. D. O'Brien, An open-label, non-randomized study of the pharmacokinetics of the nutritional supplement nicotinamide riboside (NR) and its effects on blood $NAD^+$ levels in healthy volunteers. *PLOS ONE* **12**, e0186459 (2017).

51. A. H. Kuerec, W. Wang, L. Yi, R. Tao, Z. Lin, A. Vaidya, S. Pendse, S. Thasma, N. Andhalkar, G. Avhad, V. Kumbhar, A. B. Maier, Towards personalized nicotinamide mononucleotide (NMN) supplementation: Nicotinamide adenine dinucleotide (NAD) concentration. *Mech. Ageing Dev.* **218**, 111917 (2024).

52. C. R. Martens, B. A. Denman, M. R. Mazzo, M. L. Armstrong, N. Reisdorph, M. B. McQueen, M. Chonchol, D. R. Seals, Chronic nicotinamide riboside supplementation is well-tolerated and elevates $NAD^+$ in healthy middle-aged and older adults. *Nat. Commun.* **9**, 1286 (2018).

53. M. E. Orr, E. Kotkowski, P. Ramirez, D. Bair-Kelps, Q. Liu, C. Brenner, M. S. Schmidt, P. T. Fox, A. Larbi, C. Tan, G. Wong, J. Gelfond, B. Frost, S. Espinoza, N. Musi, B. Powers, A randomized placebo-controlled trial of nicotinamide riboside in older adults with mild cognitive impairment. *Geroscience* **46**, 665–682 (2024).

54. B. Yulug, O. Altay, X. Li, L. Hanoglu, S. Cankaya, S. Lam, H. A. Velioglu, H. Yang, E. Coskun, E. Idil, Combined metabolic activators improve cognitive functions in Alzheimer's disease patients: A randomised, double-blinded, placebo-controlled phase-II trial. *Transl. Neurodegener.* **12**, 4 (2023).

55. C. Y. Wu, A. C. Kupferschmid, L. Chen, A. J. McManus, P. Kivisäkk, J. A. Galler, N. A. Schwab, L. A. DesRuisseaux, V. J. Williams, J. Gerber, M. Riley, C. Young, E. Guzmán-Vélez, H. H. Dodge, R. E. Tanzi, C. M. Singer, S. E. Arnold, Cognitive and Alzheimer's disease biomarker effects of oral nicotinamide riboside (NR) supplementation in older adults with subjective cognitive decline and mild cognitive impairment. *Alzheimers Dement.* **11**, e70023 (2025).

56. M. Vreones, M. Mustapic, R. Moaddel, K. A. Pucha, J. Lovett, D. R. Seals, D. Kapogiannis, C. R. Martens, Oral nicotinamide riboside raises $NAD^+$ and lowers biomarkers of neurodegenerative pathology in plasma extracellular vesicles enriched for neuronal origin. *Aging Cell* **22**, e13754 (2023).

57. H. Berven, S. Kverneng, E. Sheard, M. Søgnen, S. A. Af Geijerstam, K. Haugarvoll, G.-O. Skeie, C. Dölle, C. Tzoulis, NR-SAFE: A randomized, double-blind safety trial of high dose nicotinamide riboside in Parkinson's disease. *Nat. Commun.* **14**, 7793 (2023).

58. B. Brakedal, C. Dölle, F. Riemer, Y. Ma, G. S. Nido, G. O. Skeie, A. R. Craven, T. Schwarzlmüller, N. Brekke, J. Diab, The NADPARK study: A randomized phase I trial of nicotinamide riboside supplementation in Parkinson's disease. *Cell Metab.* **34**, 396–407.e96 (2022).

59. M. Yoshino, J. Yoshino, B. D. Kayser, G. J. Patti, M. P. Franczyk, K. F. Mills, M. Sindelar, T. Pietka, B. W. Patterson, S.-I. Imai, S. Klein, Nicotinamide mononucleotide increases muscle insulin sensitivity in prediabetic women. *Science* **372**, 1224–1229 (2021).

60. M. Kim, J. Seol, T. Sato, Y. Fukamizu, T. Sakurai, T. Okura, Effect of 12-week intake of nicotinamide mononucleotide on sleep quality, fatigue, and physical performance in older Japanese adults: A randomized, double-blind placebo-controlled study. *Nutrients* **14**, 755 (2022).

61. H. Akasaka, H. Nakagami, K. Sugimoto, Y. Yasunobe, T. Minami, T. Fujimoto, K. Yamamoto, C. Hara, A. Shiraki, K. Nishida, K. Asano, M. Kanou, K. Yamana, S. I. Imai, H. Rakugi, Effects of nicotinamide mononucleotide on older patients with diabetes and

impaired physical performance: A prospective, placebo-controlled, double-blind study. *Geriatr. Gerontol. Int.* **23**, 38–43 (2023).

62. K. M. Pencina, R. Valderrabano, B. Wipper, A. R. Orkaby, K. F. Reid, T. Storer, A. P. Lin, S. Merugumala, L. Wilson, N. Latham, C. Ghattas-Puylara, N. E. Ozimek, M. Cheng, A. Bhargava, Y. Memish-Beleva, B. Lawney, S. Lavu, P. M. Swain, R. S. Apte, D. A. Sinclair, D. Livingston, S. Bhasin, Nicotinamide adenine dinucleotide augmentation in overweight or obese middle-aged and older adults: A physiologic study. *J. Clin. Endocrinol. Metab.* **108**, 1968–1980 (2023).

63. K. M. Pencina, S. Lavu, M. Dos Santos, Y. M. Beleva, M. Cheng, D. Livingston, S. Bhasin, MIB-626, an oral formulation of a microcrystalline unique polymorph of β-nicotinamide mononucleotide, increases circulating nicotinamide adenine dinucleotide and its metabolome in middle-aged and older adults. *J. Gerontol. A Biol. Sci. Med. Sci.* **78**, 90–96 (2023).

64. T. S. Luongo, J. M. Eller, M.-J. Lu, M. Niere, F. Raith, C. Perry, M. R. Bornstein, P. Oliphint, L. Wang, M. R. McReynolds, SLC25A51 is a mammalian mitochondrial $NAD^+$ transporter. *Nature* **588**, 174–179 (2020).

65. A. Grozio, K. F. Mills, J. Yoshino, S. Bruzzone, G. Sociali, K. Tokizane, H. C. Lei, R. Cunningham, Y. Sasaki, M. E. Migaud, S. I. Imai, Slc12a8 is a nicotinamide mononucleotide transporter. *Nat. Metab.* **1**, 47–57 (2019).

66. J. Giroud-Gerbetant, M. Joffraud, M. P. Giner, A. Cercillieux, S. Bartova, M. V. Makarov, R. Zapata-Pérez, J. L. Sánchez-García, R. H. Houtkooper, M. E. Migaud, S. Moco, C. Canto, A reduced form of nicotinamide riboside defines a new path for $NAD^+$ biosynthesis and acts as an orally bioavailable $NAD^+$ precursor. *Mol. Metab.* **30**, 192–202 (2019).

67. M. M. Murata, X. Kong, E. Moncada, Y. Chen, H. Imamura, P. Wang, M. W. Berns, K. Yokomori, M. A. Digman, $NAD^+$ consumption by PARP1 in response to DNA damage triggers metabolic shift critical for damaged cell survival. *Mol. Biol. Cell* **30**, 2584–2597 (2019).

68. C. B. Peek, A. H. Affinati, K. M. Ramsey, H. Y. Kuo, W. Yu, L. A. Sena, O. Ilkayeva, B. Marcheva, Y. Kobayashi, C. Omura, D. C. Levine, D. J. Bacsik, D. Gius, C. B. Newgard, E. Goetzman, N. S. Chandel, J. M. Denu, M. Mrksich, J. Bass, Circadian clock $NAD^+$ cycle drives mitochondrial oxidative metabolism in mice. *Science* **342**, 1243417 (2013).

69. A. Chiarugi, C. Dölle, R. Felici, M. Ziegler, The NAD metabolome—A key determinant of cancer cell biology. *Nat. Rev. Cancer* **12**, 741–752 (2012).

70. J. da Veiga Moreira, S. Peres, J.-M. Steyaert, E. Bigan, L. Paulevé, M. L. Nogueira, L. Schwartz, Cell cycle progression is regulated by intertwined redox oscillators. *Theor. Biol. Med. Model.* **12**, 10 (2015).

71. C. H. Serezani, M. N. Ballinger, D. M. Aronoff, M. Peters-Golden, Cyclic AMP: Master regulator of innate immune cell function. *Am. J. Respir. Cell Mol. Biol.* **39**, 127–132 (2008).

72. N. G. Norwitz, S. Sethi, C. M. Palmer, Ketogenic diet as a metabolic treatment for mental illness. *Curr. Opin. Endocrinol. Diabetes Obes.* **27**, 269–274 (2020).

73. Z. Jiang, X. Yin, M. Wang, T. Chen, Y. Wang, Z. Gao, Z. Wang, Effects of ketogenic diet on neuroinflammation in neurodegenerative diseases. *Aging Dis.* **13**, 1146–1165 (2022).

74. J. Anderson, E. Ozan, V.-A. Chouinard, G. Grant, A. MacDonald, L. Thakkar, C. Palmer, The ketogenic diet as a transdiagnostic treatment for neuropsychiatric disorders: Mechanisms and clinical outcomes. *Curr. Treat. Options Psych.* **12**, 1 (2025).

75. S. Sethi, J. M. Ford, The role of ketogenic metabolic therapy on the brain in serious mental illness: A review. *J. Psychiatr. Brain Sci.* **7**, e220009 (2022).

76. M. Fortier, C.-A. Castellano, E. Croteau, F. Langlois, C. Bocti, V. St-Pierre, C. Vandenberghe, M. Bernier, M. Roy, M. Descoteaux, K. Whittingstall, M. Lepage, É. E. Turcotte, T. Fulop, S. C. Cunnane, A ketogenic drink improves brain energy and some measures of cognition in mild cognitive impairment. *Alzheimers Dement.* **15**, 625–634 (2019).

77. M. Roy, M. Edde, M. Fortier, E. Croteau, C.-A. Castellano, V. St-Pierre, C. Vandenberghe, F. Rheault, M. Dadar, S. Duchesne, C. Bocti, T. Fulop, S. C. Cunnane, M. Descoteaux, A ketogenic intervention improves dorsal attention network functional and structural connectivity in mild cognitive impairment. *Neurobiol. Aging* **115**, 77–87 (2022).

78. M. Fortier, C. A. Castellano, V. St-Pierre, É. Myette-Côté, F. Langlois, M. Roy, M. C. Morin, C. Bocti, T. Fulop, J. P. Godin, C. Delannoy, B. Cuenoud, S. C. Cunnane, A ketogenic drink improves cognition in mild cognitive impairment: Results of a 6-month RCT. *Alzheimers Dement.* **17**, 543–552 (2021).

79. E. Croteau, C.-A. Castellano, M. A. Richard, M. Fortier, S. Nugent, M. Lepage, S. Duchesne, K. Whittingstall, É. E. Turcotte, C. Bocti, Ketogenic medium chain triglycerides increase brain energy metabolism in Alzheimer's disease. *J Alzheimer's Dis* **64**, 551–561 (2018).

80. S. T. Henderson, J. L. Vogel, L. J. Barr, F. Garvin, J. J. Jones, L. C. Costantini, Study of the ketogenic agent AC-1202 in mild to moderate Alzheimer's disease: A randomized, double-blind, placebo-controlled, multicenter trial. *Nutr. Metab.* **6**, 31 (2009).

81. A. Kumar, M. Sharma, Y. Su, S. Singh, F.-C. Hsu, B. J. Neth, T. C. Register, K. Blennow, H. Zetterberg, S. Craft, G. Deep, Small extracellular vesicles in plasma reveal molecular effects of modified Mediterranean-ketogenic diet in participants with mild cognitive impairment. *Brain Commun.* **4**, fcac262 (2022).

82.. Myette-Côté, V. St-Pierre, S. Beaulieu, C.-A. Castellano, M. Fortier, M. Plourde, C. Bocti, T. Fulop, S. C. Cunnane, The effect of a 6-month ketogenic medium-chain triglyceride supplement on plasma cardiometabolic and inflammatory markers in mild cognitive impairment. *Prostaglandins Leukot. Essent. Fatty Acids* **169**, 102236 (2021).

83. A. H. Choi, M. Delgado, K. Y. Chen, S. T. Chung, A. Courville, S. A. Turner, S. Yang, K. Airaghi, I. Dustin, P. McGurrin, T. Wu, M. Hallett, D. J. Ehrlich, A randomized feasibility trial of medium chain triglyceride-supplemented ketogenic diet in people with Parkinson's disease. *BMC Neurol.* **24**, 106 (2024).

84. M. M. Tidman, D. White, T. White, Effects of an low carbohydrate/healthy fat/ketogenic diet on biomarkers of health and symptoms, anxiety and depression in Parkinson's disease: A pilot study. *Neurodegener. Dis. Manag.* **12**, 57–66 (2022).

85. H. Koyuncu, V. Fidan, H. Toktas, O. Binay, H. Celik, Effect of ketogenic diet versus regular diet on voice quality of patients with Parkinson's disease. *Acta Neurol. Belg.* **121**, 1729–1732 (2021).

86. M. C. Phillips, D. K. Murtagh, L. J. Gilbertson, F. J. Asztely, C. D. Lynch, Low-fat versus ketogenic diet in Parkinson's disease: A pilot randomized controlled trial. *Mov. Disord.* **33**, 1306–1314 (2018).

87. S. Sethi, D. Wakeham, T. Ketter, F. Hooshmand, J. Bjornstad, B. Richards, E. Westman, R. M. Krauss, L. Saslow, Ketogenic diet intervention on metabolic and psychiatric health in bipolar and schizophrenia: A pilot trial. *Psychiatry Res.* **335**, 115866 (2024).

88. I. H. Campbell, N. Needham, H. Grossi, I. Kamenska, S. Luz, S. Sheehan, G. Thompson, M. J. Thrippleton, M. C. Gibbs, J. Leitao, T. Moses, K. Burgess, B. P. Rigby, S. A. Simpson, E. McIntosh, R. Brown, B. Meadowcroft, F. Creasy, M. Mitchell-Grigorjeva, J. Norrie, A. McLellan, C. Fisher, T. Zieliński, G. Gaggioni, H. Campbell, D. J. Smith, A pilot study of a ketogenic diet in bipolar disorder: Clinical, metabolic and magnetic resonance spectroscopy findings. *BJPsych Open* **11**, e34 (2025).

89. A. Danan, E. C. Westman, L. R. Saslow, G. Ede, The ketogenic diet for refractory mental illness: A retrospective analysis of 31 inpatients. *Front. Psych.* **13**, 951376 (2022).

90. R. G. Popescu, A. Dinischiotu, T. Soare, E. Vlase, G. C. Marinescu, Nicotinamide mononucleotide (NMN) works in type 2 diabetes through unexpected effects in adipose tissue, not by mitochondrial biogenesis. *Int. J. Mol. Sci.* **25**, 2594 (2024).

91. R. S. Fletcher, G. G. Lavery, The emergence of the nicotinamide riboside kinases in the regulation of $NAD^+$ metabolism. *J. Mol. Endocrinol.* **61**, R107–R121 (2018).

92. M. Lu, X. H. Zhu, W. Chen, In vivo $^{31}$P MRS assessment of intracellular NAD metabolites and $NAD^+$/NADH redox state in human brain at 4 T. *NMR Biomed.* **29**, 1010–1017 (2016).

93. R. Guo, S. Yang, H. M. Wiesner, Y. Li, Y. Zhao, Z. P. Liang, W. Chen, X. H. Zhu, Mapping intracellular NAD content in entire human brain using phosphorus-31 MR spectroscopic imaging at 7 Tesla. *Front. Neurosci.* **18**, 1389111 (2024).

94. R. Skupienski, K. Q. Do, L. Xin, In vivo $^{31}$P magnetic resonance spectroscopy study of mouse cerebral NAD content and redox state during neurodevelopment. *Sci. Rep.* **10**, 15623 (2020).

95. V.-A. Chouinard, S.-Y. Kim, L. Valeri, C. Yuksel, K. P. Ryan, G. Chouinard, B. M. Cohen, F. Du, D. Öngür, Brain bioenergetics and redox state measured by $^{31}$P magnetic resonance spectroscopy in unaffected siblings of patients with psychotic disorders. *Schizophr. Res.* **187**, 11–16 (2017).

96. B. Cuenoud, Z. Huang, M. Hartweg, M. Widmaier, S. Lim, D. Wenz, L. Xin, Effect of circadian rhythm on NAD and other metabolites in human brain. *Front. Physiol.* **14**, 1285776 (2023).

97. R. A. de Graaf, K. L. Behar, Detection of cerebral $NAD^+$ by in vivo $^1$H NMR spectroscopy. *NMR Biomed.* **27**, 802–809 (2014).

98. P. Bagga, H. Hariharan, N. E. Wilson, J. C. Beer, R. T. Shinohara, M. A. Elliott, J. A. Baur, F. M. Marincola, W. R. Witschey, M. Haris, J. A. Detre, R. Reddy, Single-voxel $^1$H MR spectroscopy of cerebral nicotinamide adenine dinucleotide ($NAD^+$) in humans at 7T using a 32-channel volume coil. *Magn. Reson. Med.* **83**, 806–814 (2020).

99. S. Swago, N. E. Wilson, M. A. Elliott, R. Reddy, W. R. Witschey, R. P. R. Nanga, Quantification of tryptophan and $NAD^+$ proton magnetization exchange with water using downfield 1h mrs in the human brain at 7 T. *Magn. Reson. Med.* **95**, 1336–1344 (2025).

100. R. P. R. Nanga, C. E. Wiers, M. A. Elliott, N. E. Wilson, F. Liu, Q. Cao, S. Swago, P. S. Jacobs, R. Armbruster, D. Reddy, J. A. Baur, W. R. Witschey, J. A. Detre, R. Reddy, Acute

nicotinamide riboside supplementation increases human cerebral $NAD^+$ levels in vivo. *Magn. Reson. Med.* **92**, 2284–2293 (2024).

101. S. Y. Kim, W. Chen, D. Ongur, F. Du, Rapid and simultaneous measurement of phosphorus metabolite pool size ratio and reaction kinetics of enzymes in vivo. *J. Magn. Reson. Imaging* **47**, 210–221 (2018).

102. Q. Xiong, F. Du, X. Zhu, P. Zhang, P. Suntharalingam, J. Ippolito, F. D. Kamdar, W. Chen, J. Zhang, ATP production rate via creatine kinase or ATP synthase in vivo: A novel superfast magnetization saturation transfer method. *Circ. Res.* **108**, 653–663 (2011).

103. F. Du, X. H. Zhu, H. Qiao, X. Zhang, W. Chen, Efficient in vivo 31P magnetization transfer approach for noninvasively determining multiple kinetic parameters and metabolic fluxes of ATP metabolism in the human brain. *Magn. Reson. Med.* **57**, 103–114 (2007).

104. C. Yuksel, F. Du, C. Ravichandran, J. R. Goldbach, T. Thida, P. Lin, B. Dora, J. Gelda, L. O'Connor, S. Sehovic, S. Gruber, D. Ongur, B. M. Cohen, Abnormal high-energy phosphate molecule metabolism during regional brain activation in patients with bipolar disorder. *Mol. Psychiatry* **20**, 1079–1084 (2015).

105. X. H. Zhu, W. Chen, In vivo X-nuclear MRS imaging methods for quantitative assessment of neuroenergetic biomarkers in studying brain function and aging. *Front. Aging Neurosci.* **10**, 394 (2018).

106. D. Tomasi, G.-J. Wang, N. D. Volkow, Energetic cost of brain functional connectivity. *Proc. Natl. Acad. Sci. U.S.A.* **110**, 13642–13647 (2013).

107. M. E. Raichle, in *Comprehensive Physiology*, R. Terjung, Ed. (American Physiological Society, 2011), pp. 643–674.

108. O. Kann, I. E. Papageorgiou, A. Draguhn, Highly energized inhibitory interneurons are a central element for information processing in cortical networks. *J. Cereb. Blood Flow Metab.* **34**, 1270–1282 (2014).

109. X. Chen, X. Fan, Y. Hu, C. Zuo, S. Whitfield-Gabrieli, D. Holt, Q. Gong, Y. Yang, D. A. Pizzagalli, F. Du, D. Ongur, Regional GABA concentrations modulate inter-network resting-state functional connectivity. *Cereb. Cortex* **29**, 1607–1618 (2019).

110. X. Chen, X. Song, D. Ongur, F. Du, Association of default-mode network neurotransmitters and inter-network functional connectivity in first episode psychosis. *Neuropsychopharmacology* **48**, 781–788 (2023).

111. L. R. Mujica-Parodi, A. Amgalan, S. F. Sultan, B. Antal, X. Sun, S. Skiena, A. Lithen, N. Adra, E. M. Ratai, C. Weistuch, S. T. Govindarajan, H. H. Strey, K. A. Dill, S. M. Stufflebeam, R. L. Veech, K. Clarke, Diet modulates brain network stability, a biomarker for brain aging, in young adults. *Proc. Natl. Acad. Sci. U.S.A.* **117**, 6170–6177 (2020).

112. V. Demarin, D. Storga-Tomić, M. Bosnar-Puretić, I. Martinić-Popović, J. Birkmayer, in *7th Congress of the European Society for Clinical Neuropharmacology.* J. Neural Transm. (2004), pp. 41–45.

113. M. Rainer, E. Kraxberger, M. Haushofer, H. Mucke, K. Jellinger, No evidence for cognitive improvement from oral nicotinamide adenine dinucleotide (NADH) in dementia. *J. Neural Transm.* **107**, 1475–1481 (2000).

114. W. Kuhn, T. Müller, R. Winkel, S. Danielczik, A. Gerstner, R. Häcker, C. Mattern, H. Przuntek, Parenteral application of NADH in Parkinson’s disease: Clinical improvement partially due to stimulation of endogenous levodopa biosynthesis. *J. Neural Transm.* **103**, 1187–1193 (1996).

115. N. Dizdar, B. Kågedal, B. Lindvall, Treatment of Parkinson’s disease with NADH. *Acta Neurol. Scand.* **90**, 345–347 (1994).

116. J. G. Birkmayer, C. Vrecko, D. Volc, W. Birkmayer, Nicotinamide adenine dinucleotide (NADH)—A new therapeutic approach to Parkinson's disease: Comparison of oral and parenteral application. *Acta Neurol. Scand. Suppl.* **87**, 32–35 (1993).

117. W. Birkmayer, G. Birkmayer, Nicotinamidadenindinucleotide (NADH): The new approach in the therapy of Parkinson's disease. *Ann. Clin. Lab. Sci.* **19**, 38–43 (1989).

118. H. Meltzer, R. Shader, L. Grinspoon, The behavioral effects of nicotinamide adenine dinucleotide in chronic schizophrenia. *Psychopharmacologia* **15**, 144–152 (1969).

119. N. Kline, G. Barclay, J. Cole, A. Esser, H. Lehmann, J. Wittenborn, Controlled evaluation of nicotinamide adenine dinucleotide in the treatment of chronic schizophrenic patients. *Br. J. Psychiatry* **113**, 731–742 (1967).

120. W. R. Ashby, G. Collins, M. Bassett, The effects of nicotinic acid, nicotinamide, and placebo on the chronic schizophrenic. *J. Ment. Sci.* **106**, 1555–1559 (1960).

121. J. B. Jensen, O. L. Dollerup, A. B. Møller, T. B. Billeskov, E. Dalbram, S. Chubanava, M. V. Damgaard, R. W. Dellinger, K. Trošt, T. Moritz, S. Ringgaard, N. Møller, J. T. Treebak, J. Farup, N. Jessen, A randomized placebo-controlled trial of nicotinamide riboside and pterostilbene supplementation in experimental muscle injury in elderly individuals. *JCI Insight* **7**, e158314 (2022).

122. Y. S. Elhassan, K. Kluckova, R. S. Fletcher, M. S. Schmidt, A. Garten, C. L. Doig, D. M. Cartwright, L. Oakey, C. V. Burley, N. Jenkinson, Nicotinamide riboside augments the aged human skeletal muscle $NAD^+$ metabolome and induces transcriptomic and anti-inflammatory signatures. *Cell Rep.* **28**, 1717–1728.e16 (2019).

123. M. Morifuji, S. Higashi, S. Ebihara, M. Nagata, Ingestion of β-nicotinamide mononucleotide increased blood NAD levels, maintained walking speed, and improved sleep quality in older adults in a double-blind randomized, placebo-controlled study. *GeroScience* **46**, 4671–4688 (2024).

124. T. Yamane, M. Imai, T. Bamba, S. Uchiyama, Nicotinamide mononucleotide (NMN) intake increases plasma NMN and insulin levels in healthy subjects. *Clin. Nutr. ESPEN* **56**, 83–86 (2023).

125. M. Igarashi, Y. Nakagawa-Nagahama, M. Miura, K. Kashiwabara, K. Yaku, M. Sawada, R. Sekine, Y. Fukamizu, T. Sato, T. Sakurai, J. Sato, K. Ino, N. Kubota, T. Nakagawa, T. Kadowaki, T. Yamauchi, Chronic nicotinamide mononucleotide supplementation elevates blood nicotinamide adenine dinucleotide levels and alters muscle function in healthy older men. *Aging* **8**, 5 (2022).

126. K. Okabe, K. Yaku, Y. Uchida, Y. Fukamizu, T. Sato, T. Sakurai, K. Tobe, T. Nakagawa, Oral administration of nicotinamide mononucleotide is safe and efficiently increases blood nicotinamide adenine dinucleotide levels in healthy subjects. *Front. Nutr.* **9**, 868640 (2022).

127. H. Huang, A multicentre, randomised, double blind, parallel design, placebo controlled study to evaluate the efficacy and safety of uthever (NMN supplement), an orally administered supplementation in middle aged and older adults. *Front. Aging* **3**, 851698 (2022).

128. S. Kimura, M. Ichikawa, S. Sugawara, T. Katagiri, Y. Hirasawa, T. Ishikawa, W. Matsunaga, A. Gotoh, Intravenous uptake of NMN is safely metabolize and increases $NAD^+$ levels in healthy subjects (2022). https://doi.org/10.21203/rs.3.rs-1298321/v1.

129. Y. Fukamizu, Y. Uchida, A. Shigekawa, T. Sato, H. Kosaka, T. Sakurai, Safety evaluation of β-nicotinamide mononucleotide oral administration in healthy adult men and women. *Sci. Rep.* **12**, 14442 (2022).

130. J. Irie, E. Inagaki, M. Fujita, H. Nakaya, M. Mitsuishi, S. Yamaguchi, K. Yamashita, S. Shigaki, T. Ono, H. Yukioka, H. Okano, Y. I. Nabeshima, S. I. Imai, M. Yasui, K. Tsubota, H. Itoh, Effect of oral administration of nicotinamide mononucleotide on clinical parameters and nicotinamide metabolite levels in healthy Japanese men. *Endocr. J.* **67**, 153–160 (2020).

131. A. Buchholz, P. Deme, J. F. Betz, J. Brandt, N. Haughey, M. C. Cervenka, A randomized feasibility trial of the modified Atkins diet in older adults with mild cognitive impairment due to Alzheimer's disease. *Front. Endocrinol.* **15**, 1182519 (2024).

132. A. J. Hanson, W. A. Banks, L. F. Bettcher, R. Pepin, D. Raftery, S. L. Navarro, S. Craft, Cerebrospinal fluid metabolomics: Pilot study of using metabolomics to assess diet and metabolic interventions in Alzheimer's disease and mild cognitive impairment. *Metabolites* **13**, 569 (2023).

133. M. C. L. Phillips, L. M. Deprez, G. M. N. Mortimer, D. K. J. Murtagh, S. McCoy, R. Mylchreest, L. J. Gilbertson, K. M. Clark, P. V. Simpson, E. J. McManus, J.-E. Oh, S. Yadavaraj, V. M. King, A. Pillai, B. Romero-Ferrando, M. Brinkhuis, B. M. Copeland, S. Samad, S. Liao, J. A. C. Schepel, Randomized crossover trial of a modified ketogenic diet in Alzheimer's disease. *Alzheimer's Res Ther* **13**, 51 (2021).

134. M. Roy, M. Fortier, F. Rheault, M. Edde, E. Croteau, C. A. Castellano, F. Langlois, V. St-Pierre, B. Cuenoud, C. Bocti, T. Fulop, M. Descoteaux, S. C. Cunnane, A ketogenic supplement improves white matter energy supply and processing speed in mild cognitive impairment. *Alzheimers Dement.* **7**, e12217 (2021).

135. M. Ota, J. Matsuo, I. Ishida, H. Takano, Y. Yokoi, H. Hori, S. Yoshida, K. Ashida, K. Nakamura, T. Takahashi, H. Kunugi, Effects of a medium-chain triglyceride-based ketogenic formula on cognitive function in patients with mild-to-moderate Alzheimer's disease. *Neurosci. Lett.* **690**, 232–236 (2019).

136. N. Torosyan, C. Sethanandha, J. D. Grill, M. L. Dilley, J. Lee, J. L. Cummings, C. Ossinalde, D. H. Silverman, Changes in regional cerebral blood flow associated with a 45 day course of the ketogenic agent, caprylidene, in patients with mild to moderate Alzheimer's disease: Results of a randomized, double-blinded, pilot study. *Exp. Gerontol.* **111**, 118–121 (2018).

137. A. Pacheco, W. Easterling, M. W. Pryer, A pilot study of the ketogenic diet in schizophrenia. *Am. J. Psychiatry* **121**, 1110–1111 (1965).

138. I. Chmiel, Ketogenic diet in therapy of bipolar affective disorder—Case report and literature review. *Psychiatr. Pol.* **56**, 1345–1363 (2022).

139. M. Saraga, N. Misson, E. Cattani, Ketogenic diet in bipolar disorder. *Bipolar Disord.* **22**, 765 (2020).

140. C. M. Palmer, J. Gilbert-Jaramillo, E. C. Westman, The ketogenic diet and remission of psychotic symptoms in schizophrenia: Two case studies. *Schizophr. Res.* **208**, 439–440 (2019).

141. J. Gilbert-Jaramillo, D. Vargas-Pico, T. Espinosa-Mendoza, S. Falk, K. Llanos-Fernández, J. Guerrero-Haro, C. Orellana-Román, C. Poveda-Loor, J. Valdevila-Figueira, C. Palmer, The effects of the ketogenic diet on psychiatric symptomatology, weight and metabolic dysfunction in schizophrenia patients. *Clin. Nutr. Metab.* **1**, 1–5 (2018).

142. C. M. Palmer, Ketogenic diet in the treatment of schizoaffective disorder: Two case studies. *Schizophr. Res.* **189**, 208–209 (2017).

143. J. R. Phelps, S. V. Siemers, R. S. El-Mallakh, The ketogenic diet for type II bipolar disorder. *Neurocase* **19**, 423–426 (2013).

144. B. D. Kraft, E. C. Westman, Schizophrenia, gluten, and low-carbohydrate, ketogenic diets: A case report and review of the literature. *Nutr. Metab.* **6**, 10 (2009).

145. Y. Yaroslavsky, Z. Stahl, R. H. Belmaker, Ketogenic diet in bipolar illness. *Bipolar Disord.* **4**, 75–75 (2002).

146. H. A. K. Lapatto, M. Kuusela, A. Heikkinen, M. Muniandy, B. W. van der Kolk, S. Gopalakrishnan, N. Pöllänen, M. Sandvik, M. S. Schmidt, S. Heinonen, S. Saari, J. Kuula, A. Hakkarainen, J. Tampio, T. Saarinen, M. R. Taskinen, N. Lundbom, P. H. Groop, M. Tiirola, P. Katajisto, M. Lehtonen, C. Brenner, J. Kaprio, S. Pekkala, M. Ollikainen, K. H. Pietiläinen, E. Pirinen, Nicotinamide riboside improves muscle mitochondrial biogenesis, satellite cell differentiation, and gut microbiota in a twin study. *Sci. Adv.* **9**, eadd5163 (2023).

147. J. Wu, K. Singh, A. Lin, A. M. Meadows, K. Wu, V. Shing, M. Bley, S. Hassanzadeh, R. D. Huffstutler, M. S. Schmidt, L. P. Blanco, R. Tian, C. Brenner, M. Pirooznia, M. J. Kaplan,

M. N. Sack, Boosting NAD$^+$ blunts TLR4-induced type I IFN in control and systemic lupus erythematosus monocytes. *J. Clin. Invest.* **132**, e139828 (2022).

148. S. Yamaguchi, J. Irie, M. Mitsuishi, Y. Uchino, H. Nakaya, R. Takemura, E. Inagaki, S. Kosugi, H. Okano, M. Yasui, K. Tsubota, K. Hayashi, J. Yoshino, H. Itoh, Safety and efficacy of long-term nicotinamide mononucleotide supplementation on metabolism, sleep, and nicotinamide adenine dinucleotide biosynthesis in healthy, middle-aged Japanese men. *Endocr. J.* **71**, 153–169 (2024).

149. T. Katayoshi, S. Uehata, N. Nakashima, T. Nakajo, N. Kitajima, M. Kageyama, K. Tsuji-Naito, Nicotinamide adenine dinucleotide metabolism and arterial stiffness after long-term nicotinamide mononucleotide supplementation: A randomized, double-blind, placebo-controlled trial. *Sci. Rep.* **13**, 2786 (2023).

150. L. Yi, A. B. Maier, R. Tao, Z. Lin, A. Vaidya, S. Pendse, S. Thasma, N. Andhalkar, G. Avhad, V. Kumbhar, The efficacy and safety of β-nicotinamide mononucleotide (NMN) supplementation in healthy middle-aged adults: A randomized, multicenter, double-blind, placebo-controlled, parallel-group, dose-dependent clinical trial. *Geroscience* **45**, 29–43 (2023).

151. M. K. Taylor, D. K. Sullivan, J. D. Mahnken, J. M. Burns, R. H. Swerdlow, Feasibility and efficacy data from a ketogenic diet intervention in Alzheimer's disease. *Alzheimers Dement.* **4**, 28–36 (2018).

152. R. Krikorian, M. D. Shidler, K. Dangelo, S. C. Couch, S. C. Benoit, D. J. Clegg, Dietary ketosis enhances memory in mild cognitive impairment. *Neurobiol. Aging* **33**, 425.e19–425.e27 (2012).

153. T. B. Vanitallie, C. Nonas, A. Di Rocco, K. Boyar, K. Hyams, S. B. Heymsfield, Treatment of Parkinson disease with diet-induced hyperketonemia: A feasibility study. *Neurology* **64**, 728–730 (2005).
